# Supplementary material for: In vivo HSC transduction in humanized mice mediated by novel capsid-modified HDAd vectors
Source: Mol Ther Methods Clin Dev. 2025 Mar 14;33(2):101448. doi: 10.1016/j.omtm.2025.101448 (PMC11995070; doi:10.1016/j.omtm.2025.101448)
Supplement: Document S1. Figures S1–S8 and Table S1 [file mmc1.pdf]

OMTM, Volume *33*

Supplemental information

In vivo HSC transduction in humanized mice mediated by novel capsid-modified HDAd vectors

Aphrodite Georgakopoulou, Hongjie Wang, Jiho Kim, Chang Li, and André Lieber

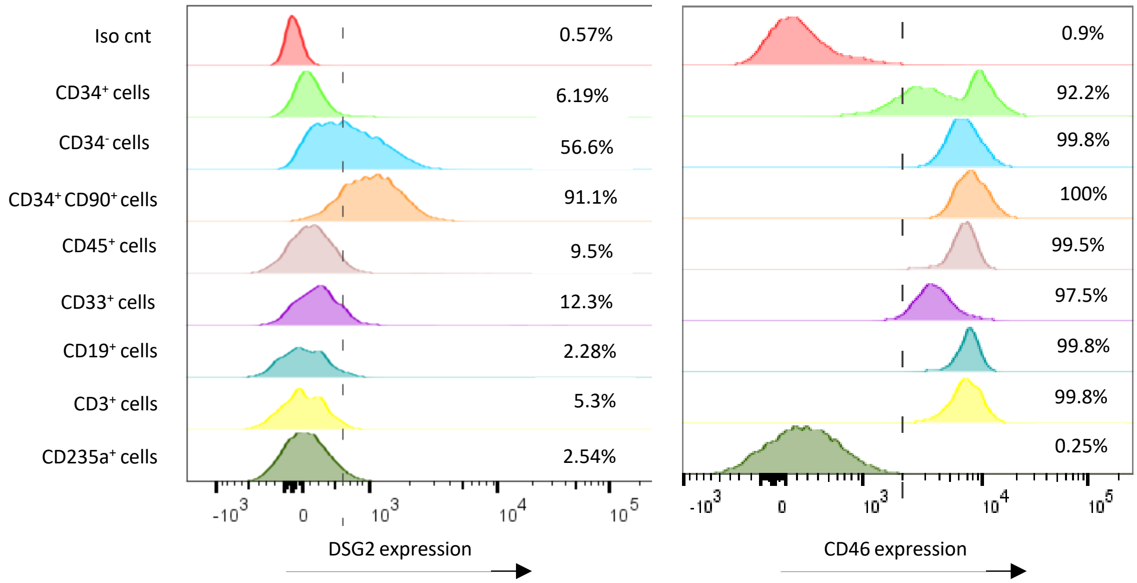

Fig.S1. Expression of HDAd attachment receptors CD46 and DSG2. Representative histograms of DSG2 and CD46 expression in human HSPCs, one day post thawing, in $CD34^-$ cell subset and several human lineages. All plots represent data from 4 different donors. Data are shown as means ± SEM. *$p \leq 0.05$ (One-way ANOVA with Bonferroni correction).

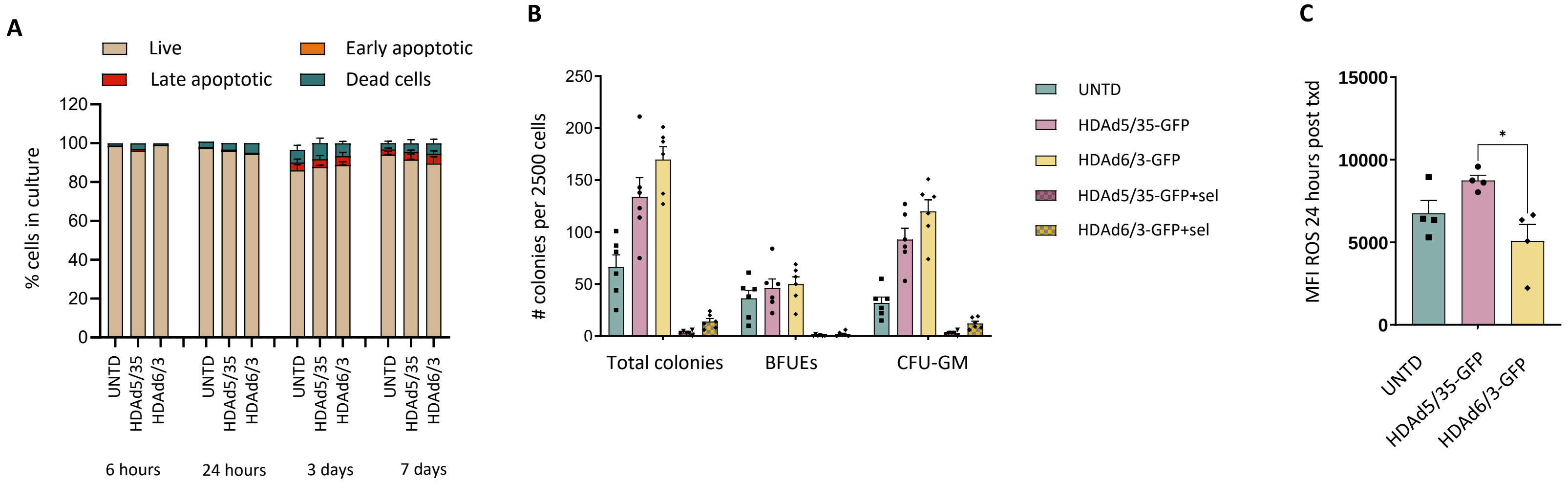

Fig.S2. Evaluation of HDAd6/3_GFP transduction efficiency in human CD34⁺ cells. A) Viability of human CD34⁺ cells 6h-, 24h-, 3 days- and 7 days- post transduction. **B)** Total colony number per 2500 human CD34⁺ cells (plated in duplicates). **C)** ROS expression 24 hours post transduction

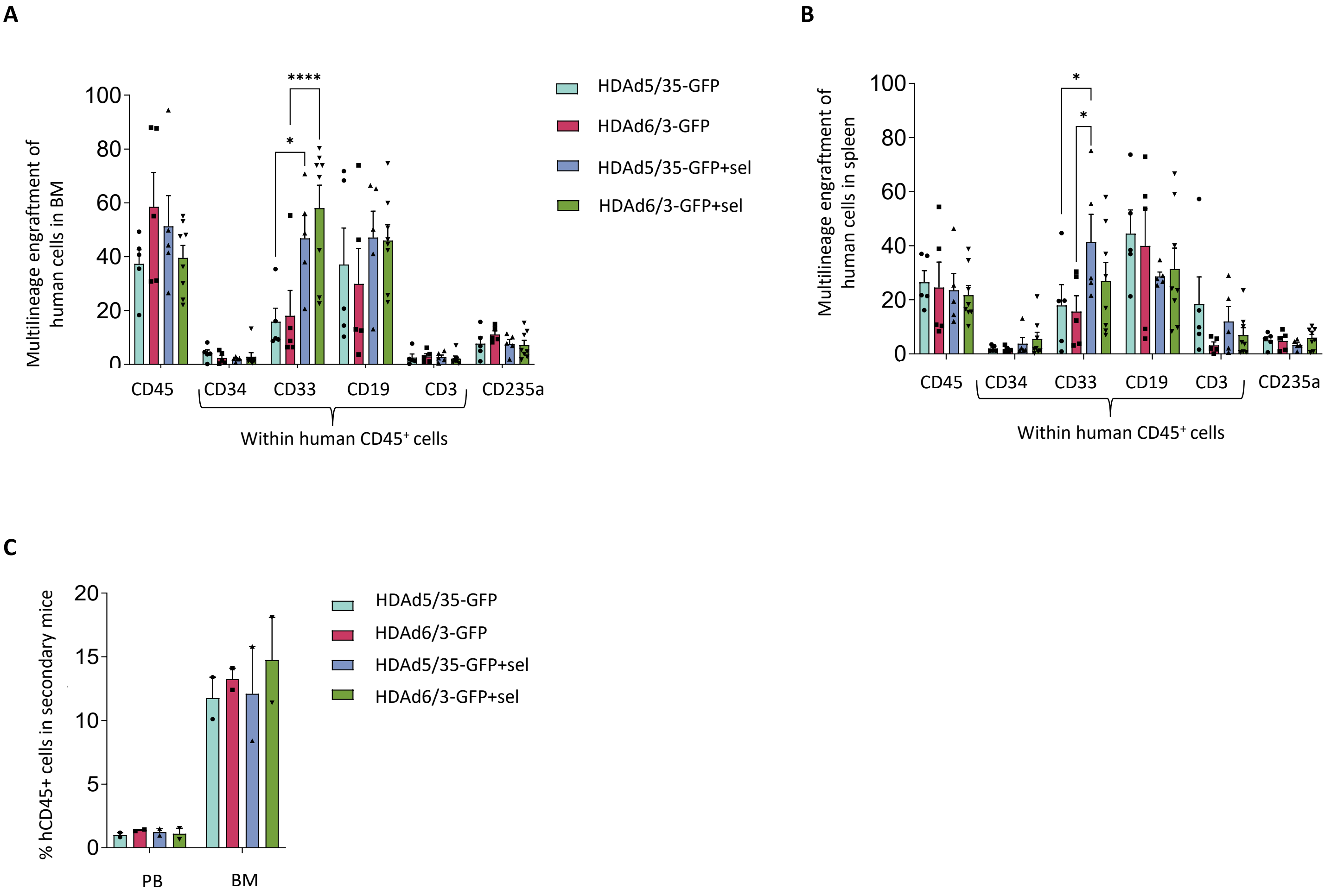

Fig.S3. Multilineage engraftment of human cells in NSGW41 mice post *in vivo* transduction with HDAd5/35-GFP or HDAd6/3-GFP. A) Multi-lineage engraftment of human cells in BM and **B)** spleen. Each symbol represents an individual mouse (HDAd5/35-GFP: N=5, HDAd6/3-GFP: N=5, HDAd5/35-GFP+sel: N=5, HDAd6/3-GFP+sel: N=8). **C)** Human chimerism in the peripheral blood and bone marrow in secondary mice. (N=8; HDAd5/35-GFP: N=2, HDAd6/3-GFP: N=2, HDAd5/35-GFP+sel: N=2 and HDAd6/3-GFP+sel: N=2). Data are shown as means ± SEM. ****p≤0.0001, *p≤0.05 (Two-way ANOVA with Bonferroni correction).

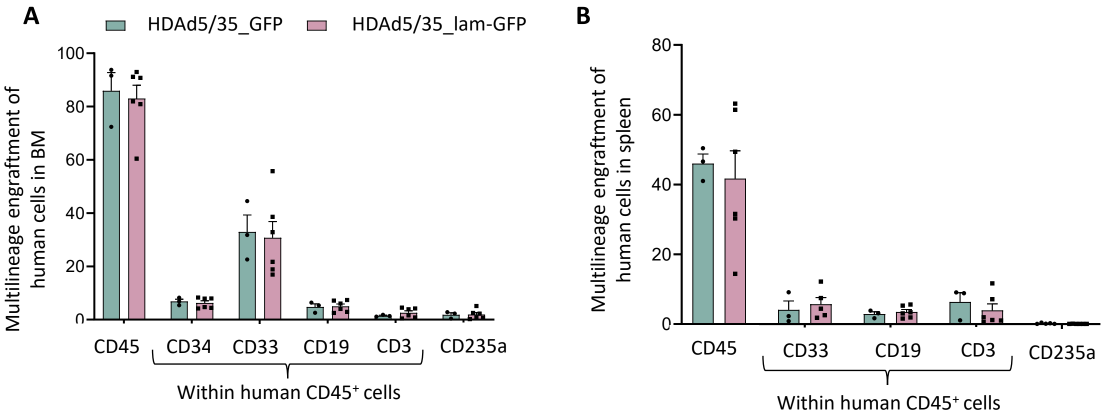

Fig.S4. Transduction efficiency of HDAd5/35_lam-GFP in human CD34$^+$ cells in humanized mice, 3 days post *in vivo* transduction. Composition of **A)** BM and **B)** Spleen. Each symbol represents an individual mouse (HDAd5/35-GFP: N=3 and HDAd5/35_lam-GFP: N=6). Data are shown as means ± SEM.

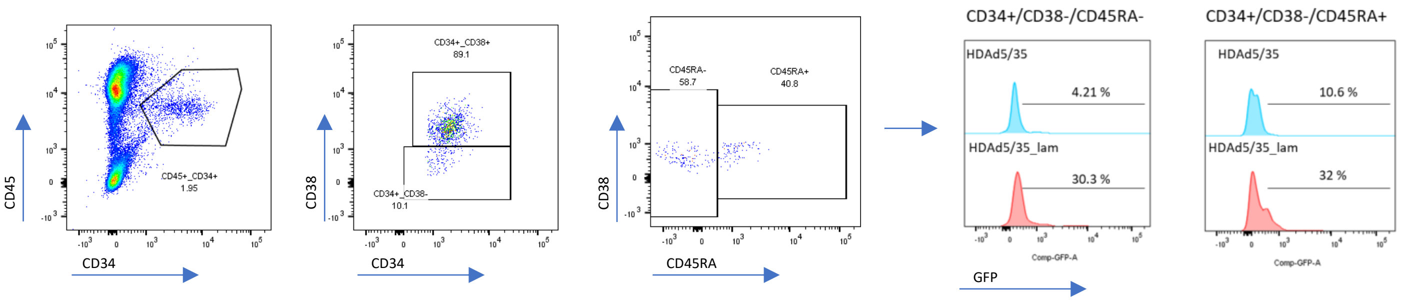

Fig.S5. Gating strategy for measuring GFP expression in HSPCs primitive subpopulations in the bone marrow of humanized mice, 3 days post *in vivo* transduction with either HDAd5/35_GFP or HDAd5/35_lam/GFP.

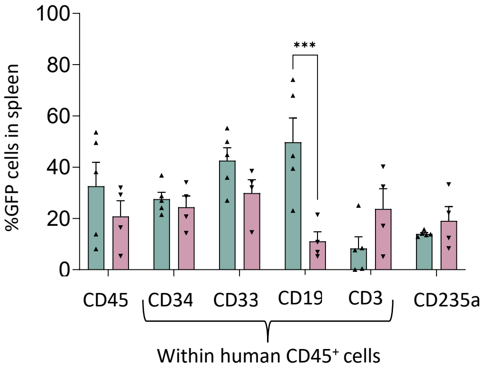

Fig.S6. Transduction efficiency of HDAd5/35_lam-GFP in hCD34⁺ cells in humanized mice, at week 15 post *in vivo* transduction. Percentage of GFP expression in human subpopulations in spleen. Each symbol represents an individual mouse (HDAd5/35-GFP: N=3 and HDAd5/35_lam-GFP: N=6). Data are shown as means ± SEM.

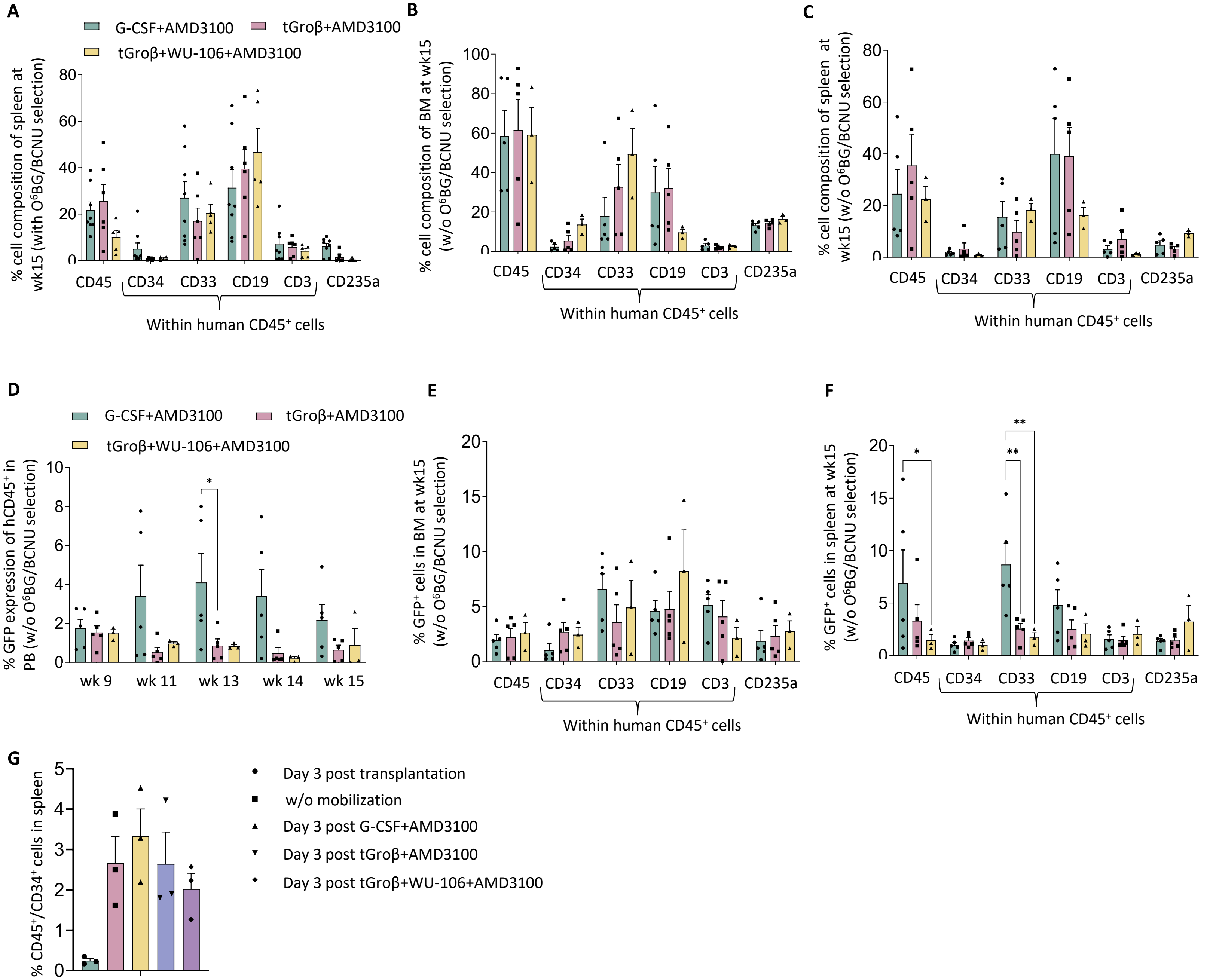

Fig.S7. *In vivo* transduction efficiency of human HSPCs with HDAd6/3-GFP mobilized with different approaches. A) Composition of human subpopulations in spleen one month after *in vivo* selection (G-CSF+AMD3100: N=8, tGroβ+AMD3100: N=6, tGroβ+WU-106+AMD3100: N=5). **Upper panel B-C)** Multilineage engraftment of human cells in **B)** BM and **C)** spleen, 8 weeks post *in vivo* transduction of mobilized human cells, without administration of O^6BG/BCNU selection drugs. **Lower panel D-F)** Percentage of GFP expressing cells in the hematopoietic tissues of mice without *in vivo* selection at week 15, two months after *in vivo* transduction of human cells **D)** in human $CD45^+$ cell in PB, **E)** in human subpopulations in BM, **F)** in human subpopulations in spleen. Groups without selection: G-CSF+AMD3100: N=5, tGroβ+AMD3100: N=5, tGroβ+WU-106+AMD3100: N=3. **G)** Assessment of homing of $CD34^+$ cells in spleen. Percentage of $CD34^+$ cells in spleen 3 days post transplantation of human $CD34^+$ cells, at week 6 without mobilization and 3 days post different mobilization approaches (N=3 mice per group). Each symbol represents an individual mouse. Data are shown as means ± SEM. **$p \leq 0.01$, *$p \leq 0.05$ (Two-way ANOVA with Bonferroni correction).

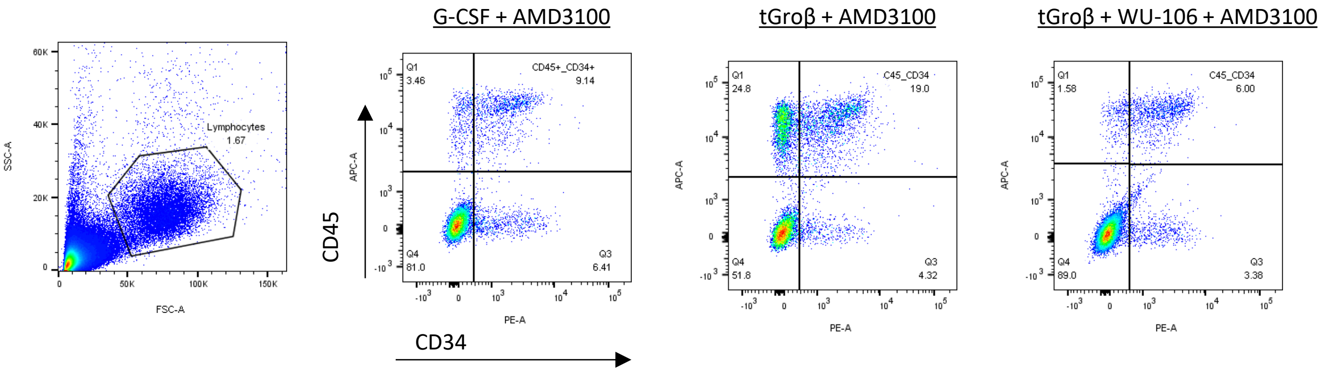

Fig.S8. A) Gating strategy for measuring the mobilization efficiency in the peripheral blood in humanized mice, at the peak of mobilization for the three mobilization schemes and representative plots of human HSCs in peripheral blood post mobilization.

Table S1. List of antibodies used for flow cytometry.

Epitope	Clone	Company	Catalog Number
**CD33**	P67.6	BD Biosciences	3058564
**CD34**	561_APC	BioLegend	343608
**CD34**	561_PE	BioLegend	343606
**CD38**	HIT2	BD Biosciences	555460
**CD90**	5E10	BD Biosciences	555597
**CD235a**	GA-R2	BD Biosciences	555570
**CD71**	M-A712	BD Biosciences	555536
**CD45**	HI30	BD Biosciences	555485
**CD19**	HIB19	BioLegend	302228
**CD3**	UCHT1	BioLegend	300406
**CD45RA**	5H9	BD Biosciences	561212
**CD46**	MEM-258	BioLegend	315304
**DSG2**	CSTEM28	ThermoFisher Scientific	12-9159-42
