# Supplementary material for: In vivo HSC transduction in humanized mice mediated by novel capsid-modified HDAd vectors
Source: Mol Ther Methods Clin Dev. 2025 Mar 14;33(2):101448. doi: 10.1016/j.omtm.2025.101448 (PMC11995070; doi:10.1016/j.omtm.2025.101448)
Supplement: Document S2. Article plus supplemental information [file mmc2.pdf]

Molecular Therapy
Methods & Clinical Development
Original Article

In vivo HSC transduction in humanized mice mediated by novel capsid-modified HDAd vectors

Aphrodite Georgakopoulou,[1] Hongjie Wang,[1] Jiho Kim,[2] Chang Li,[1] and André Lieber[1]

[1]University of Washington, Department of Medicine, Division of Medical Genetics, Seattle, WA 98195, USA; [2]PAI Life Sciences, Seattle, WA 98102, USA

We developed an *in vivo* hematopoietic stem cell (HSC) gene therapy approach consisting of HSC mobilization and intravenous injection of helper dependent adenovirus (HDAd) vectors. While we have demonstrated safety and efficacy of the *in vivo* approach in CD46-transgenic mice and rhesus macaques, studies in mice with a humanized hematopoietic system could facilitate its potential clinical translation for the treatment of hemoglobinopathies and HIV. Using mild myelo-conditioning in NSGW41 mice and cryopreserved human CD34$^+$ cells from healthy donors we achieved ∼10% human chimerism in peripheral blood. Engrafted primitive human CD45$^+$/CD34$^+$/CD90$^+$-HSCs efficiently mobilized by different approaches involving AMD3100 in combination with granulocyte colony-stimulating factor G-CSF, truncated Groβ (tGroβ), or WU106/tGroβ. At the peak of mobilization, integrating HDAd-GFP vectors were injected intravenously followed by O^{6}BG/BCNU *in vivo* selection. Long-term stable GFP expression was shown for HDAd5/35 and the new vector platforms HDAd6/3 and HDAd5/35_lam, a fiber/penton-modified vector. Two months post transduction, GFP marking in the periphery were 22.38% (8.17%), 41.12% (10.62%), and 32.15% (4.49%) for HDAd5/35, HDAd6/3, and HDAd5/35_lam, respectively. GFP levels in bone marrow were 33.53% (8.96%), 53.51% (6.95%), and 33.29% (5.21%) and in spleen 32.6% (9.25%), 33.75% (5.47%), and 20.79% (6.15%). Our study describes a new animal model for *in vivo* HSC transduction with HDAds, with implications for studies with other vectors.

INTRODUCTION

Our *in vivo* hematopoietic stem cell (HSC) transduction vehicle is based on modified adenovirus vectors devoid of all viral genes (helper dependent adenovirus [HDAd]), that can overcome limitations of currently used gene therapy vectors. HDAd vectors (1) are targeted to HSC receptors CD46 or desmoglein 2 (DSG2)[1–3] through their fiber protein, (2) have a payload capacity of 35 kb, which allows for the insertion of large transcriptionally regulatory elements[4] or multiple transgene cassettes,[5] (3) can be formulated as episomal vectors (e.g., for transient expression of genome editors[6,7]) or integrating vectors (e.g., by using a *Sleeping Beauty* transposase[8] or homology-dependent-repair[9]), and (4) can be produced easily at low costs at very high yields and titers because, unlike recombinant adeno-associated virus (rAAV) and lentivectors, no large-scale plasmid transfection is required.[10] To expand *in vivo* transduced HSCs, we used, up until recently, an *in vivo* selection mechanism based on a mutant O^6-methylguanine-DNA methyltransferase (mgmt^{P140K}) gene that confers resistance to O^6-Benzylguanine/Carmustine (O^6-BG/BCNU).[11–13] Most of our published data in mice and nonhuman primates (NHPs) were obtained with HDAd5/35++ vectors containing Ad5 capsids with Ad35 fibers that contained mutations to increase the affinity to CD46. More recently, we introduced more capsid modifications to HDAd vectors to increase their utility for *in vivo* HSC gene transfer. (1) We switched the serotype of HDAd vectors from Ad5 to Ad6, a serotype with lower prevalence of serum antibodies in humans.[2] Furthermore, the relative short length of the Ad6 fiber shaft together with Ad6 specific hexon structures greatly reduced hepatocyte transduction of HDAd6-based vectors in mice and NHPs.[2] In rhesus macaques, in contrast to HDAd5-based vectors, the HDAd6 vector did not induce detectable cytokine release when combined with dexamethasone + tocilizumab (IL-6 blocker) + anakinra (IL-1 blocker) prophylaxis. (2) We switched the CD46-targeting Ad35K++ fiber knob to the desmoglein 2 (DSG2)-targeting Ad3 fiber knob, which also contained affinity-enhancing mutations (HDAd5/3, HDAd6/3+). This has two major advantages in NHPs. Unlike HDAd5/35 vectors, HDAd5/3 and HDAd6/3 vectors are not sequestered by erythrocytes, which in NHPs express CD46. The HSC target, DSG2, is preferentially expressed on primitive HSCs[1] and not accessible in epithelial tissues,[14] which makes HDAd5/3 and HDAd6/3 vector more efficient and specific for *in vivo* transduction of primitive HSCs.

While the expression pattern of CD46 in NHPs and humans are similar (with the exception of erythrocytes), in mice CD46 is only expressed in the testes.[15] We therefore used human CD46 transgenic mice that mimic the CD46 expression pattern in humans, including its distribution in the hematopoietic system.[16] In human CD46-transgenic mice, after *in vivo* transduction/selection, transgene marking in all bone marrow mononuclear cells (MNCs) and peripheral blood cell (PBMC) lineages could be increased to >90%.[11] Our approach resulted in phenotypic correction in mouse disease models of thalassemia intermedia,[8] sickle cell disease,[5,17] murine hemophilia A,[18] and in the reversion of spontaneous cancer.[19]

Received 27 December 2024; accepted 11 March 2025;
https://doi.org/10.1016/j.omtm.2025.101448.

Correspondence: André Lieber, University of Washington, Seattle, WA 98195, USA.
E-mail: lieber00@uw.edu

© 2025 The Author(s). Published by Elsevier Inc. on behalf of The American Society of Gene and Cell Therapy.
This is an open access article under the CC BY license (http://creativecommons.org/licenses/by/4.0/).

The expression pattern of DSG2 is similar in humans and NHPs.[20,21] We generated human DSG2 transgenic mice by inserting ~90 kb of the human DSG2 locus into the mouse genome.[20] While human DSG2 expression in epithelial tissues adequately modeled the pattern in human tissues, DSG2 was not expressed in HSCs in these mice, which made *in vivo* HSC transduction studies with DSG2-targeting vectors impossible. Therefore, most of our *in vivo* HSC transduction studies with DSG2-targeting HDAds were, so far, performed in rhesus macaques.[22]

We also evaluated other species as potential models for HSC *in vivo* transduction with CD46-and DSG2-targeting HDAds. We isolated bone marrow cell fractions enriched for HSCs from rat, dog, and pig by magnetic activated cell sorting for c-Kit-positive cells (using the c-Kit Ligand-Stem Cell Factor conjugated to microbeads). However, after transduction with HDAd5/35-GFP and HDAd5/3-GFP vectors, we found less than 1% of GFP-positive cells in these fractions, indicating that CD46 and DSG2 in these species are different from the corresponding human and NHP receptors.

Therefore, an important tool to assess the efficacy of *in vivo* HSC transduction is humanized mouse models, generated by transplantation of human $CD34^+$ cells into immunodeficient mice. In our study, we used NSGW41 mice.[23] In engraftment experiments, nonirradiated NSGW41 mice show enhanced humanization in the bone marrow without preconditioning. Chimerism in PBMCs is less developed and in most cases is below 5%. Here, we first improved the human chimerism by mild myelo-conditioning and then demonstrated efficient HSC mobilization by various regimens. Based on this, we used humanized mice to study *in vivo* transduction of human HSCs with new HDAd vectors that have not been described before.

RESULTS

HDAd vectors

Previously, we reported the use of a new serotype 6-based vector containing affinity-enhanced Ad35 fibers (HDAd6/35) that infects HSCs *in vitro* and *in vivo* via CD46.[2] Here, we generated an Ad6-based vector containing fibers (shaft and knob) from Ad3 (HDAd6/3) (Figure 1A). Ad3 fiber knobs contained mutations that enhanced the virus's affinity to DSG2.[24,25] We also attempted to improve our HDAd5/35 vector platform by generating HDAd5/35_lam. This HDAd5/35 derivative contains, within an exposed penton loop, an $\alpha_6\beta_1$-binding laminin motif instead of the $\alpha_v/\beta_{3/5}$ binding RGD motif. SDS-polyacrylamide gel analysis of purified particles showed the presence of the main capsid proteins hexon (~108 kDa), penton base (~68 kDa) and fiber (~61 kDa) (Figure 1B). Clearly distinguishable were the dominant hexon bands. Notable were different sizes of what could be fibers and extra bands in HDAd6-based vectors around 75 kDa. Clearly, other techniques such as cryoelectron microscopy (cryo-EM) would be required to reveal detailed differences between the HDAd capsids. Dynamic light-scattering analysis suggests that HDAd6 derived particles (e.g., HDAd6/3) are larger than the Ad5-derived HDAds (HDAd5/35 and HDAd5/35_lam) Figure 1C).

An important feature that can affect the ability of HDAds for *in vivo* HSC transduction is sequestration by erythrocytes, which would greatly diminish the amount of vector that can reach the target cells. In an *in vitro* assay, involving qPCR of viral (GFP) genomes, we therefore measured the association of HDAd5/35, HDAd5/35_lam, and HDAd6/3 with human peripheral blood erythrocytes and PBMCs. As a positive control, we include an HDAd5 vector. Ad5 is known to bind to human erythrocytes via CAR.[26] CAR is expressed on human erythrocytes[26] while CD46 and DSG2 are not present.[3,27] Our HDAd genome data (Figure 1D) reflect the presence of attachment receptor on erythrocytes. HDAd5 binds; HDAd5/35, HDAd5/35_lam, and HDAd6/3 do not (the assay is performed in the presence of reactive complement). Of interest is the finding that HDAd5/35_lam and HDAd6/3 do not bind to PBMCs, in contrast to HDAd5 and HDAd5/35_lam. This is most likely due to the absence of interaction with specific $\alpha_{v\beta3/5}$ integrin on PBMCs. Overall, these binding data show that our new vectors, HDAd6/3 and HDAd5/35_lam are not sequestered by erythrocytes after intravenous injection. This is in agreement with our monkey studies using HDAd5/35 and DSG2-interacting HDAd6/3 vectors.[3]

CD46 is the primary attachment receptor for HDAd5/35 and HDAd5/35_lam. HDAd6/3 uses DSG2 for attachment to cells. Our flow cytometry studies demonstrated that CD46 and DSG2 are expressed at high levels on primitive human HSCs such as $CD34^+$/$CD90^+$ cells[22] (Figure S1). This is in agreement with analyses of single-cell RNA sequencing (RNA-seq) transcriptomics done by Atasheva et al., which revealed that that CD46 is widely expressed in $CD34^+$ cells whereas DSG2 expression is largely restricted to a population that is enriched for primitive HSCs.[1]

In vitro transduction of human $CD34^+$ cells with HDAd5/35-GFP and HDAd6/3-GFP

We first compared *in vitro* HDAd transduction, using peripheral blood (PB) $CD34^+$ cells from four granulocyte colony-stimulating factor (G-CSF)+AMD3100-mobilized healthy donors. HDAd5/35-GFP and HDAd6/3-GFP contained the same transgene cassette consisting of a ubiquitously active EF1α promoter driving a $mgmt^{P140}$/GFP cassette (the $mgmt^{P140K}$ cassette confers resistance to O^6BG/BCNU). Transduction efficacy of human $CD34^+$ cells and, importantly, more primitive human $CD34^+$/$CD38^-$/$CD90^+$ cells was significantly higher for the DSG2-targeting HDAd6/3-GFP vector than the CD46-targeting HDAd5/35-GFP vector (Figures 2A–2D). Analysis of GFP^+ cells demonstrated that HDAd6/3-GFP preferentially targeted more primitive HSCs (24 h post transduction: HDAd6/3: 17.84% [5.9%] vs. HDAd5/35: 7.79% [0.36%], $p = 0.042$) (Figure 2E). The data shown in Figures 2A–2E reflect GFP expression from episomal vector genomes because cells were incubated under conditions that suppress proliferation, which minimizes the loss of episomal genomes. Long-term GFP expression in human $CD34^+$ cells subjected to proliferation and differentiation required transgene integration by SB100x transposase, which was supplied by a coinfected HDAd-SB vector, as well as *in vitro* selection of stably integrated transgenes by O^6BG/BCNU (Figures 2F and 2G). In the first assay, after

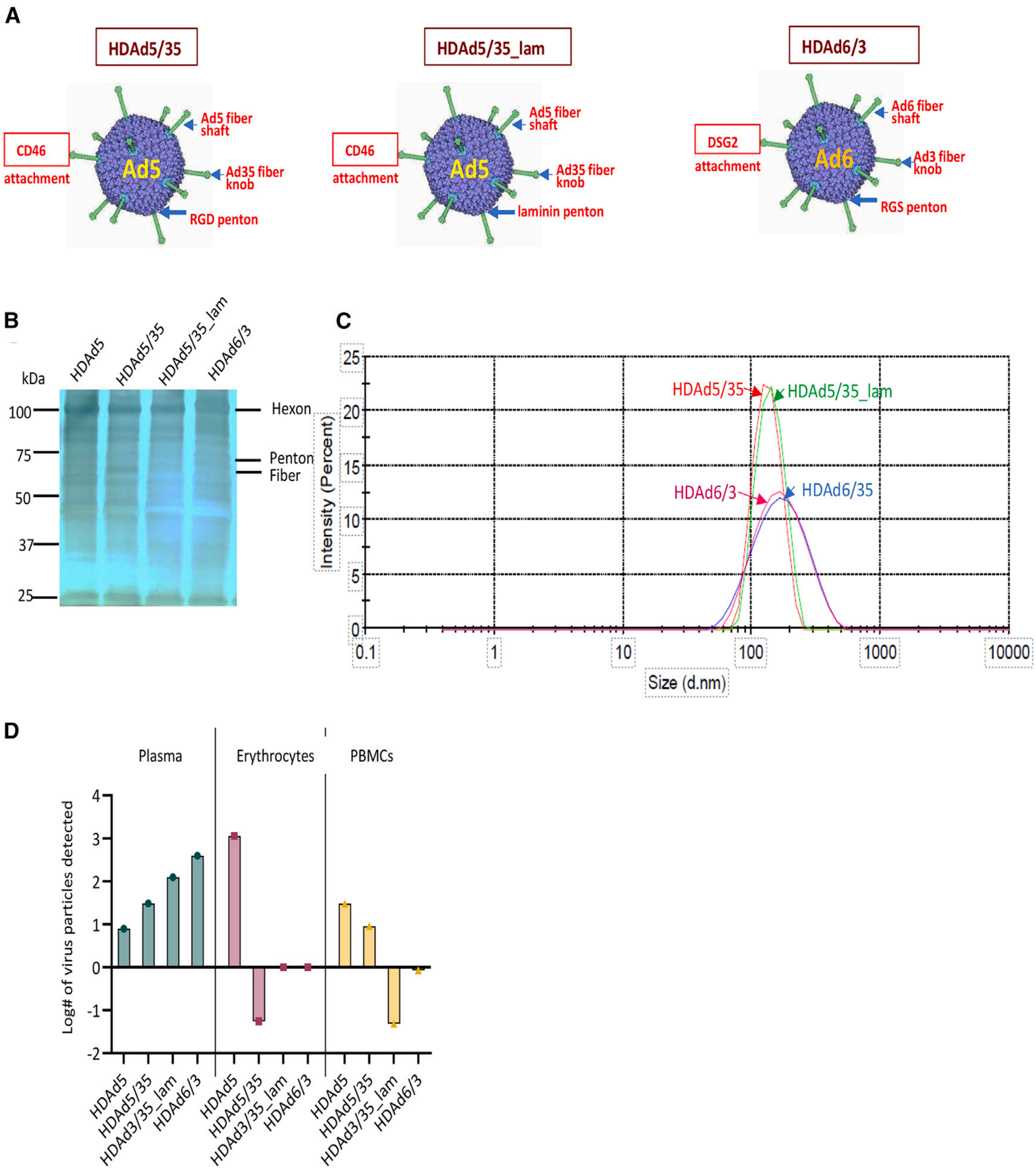

Figure 1. Characterization of HDAd particles

(A) Schematic of HDAd capsids. HDAd5/35 and HDAd5/35_lam are derived from Ad5 with the Ad5 fiber substituted by the CD46-targeting Ad35 fiber knob. HDAd5/35_lam contains an a6b1-binding laminin motif instead of the av/b3/5 binding RGD motif. HDAd6/3 is completely derived from Ad serotype 6 and contains the DSG2-binding Ad3 fiber knob. (B) SDS-polyacrylamide gel analysis of purified particles showed the presence of the main capsid proteins hexon (∼108 kDa), penton base (∼68 kDa), and fiber (∼61 kDa) with some variations among HDAd5, HDAd5/35, HDAd5/35_lam, HDAd6/35, and HDAd6/3. Clearly distinguishable were the dominant hexon bands. Notable were different sizes of what could be fibers and extra bands in HDAd6-based vectors around 75 kDa. Clearly, other techniques such as cryo-EM would be required to reveal differences between the HDAd capsids. (C) Analysis of particle size was by dynamic light scattering (DLS). The y axis shows the distribution of the intensity of the light being defracted during the DLS process. (D) Binding HDAd to human erythrocytes. HDAd5, HDAd5/35, HDAd5/35_lam, and HDAd6/3 were incubated with heparinzed blood and the number of viral genomes associated with erythrocytes, PBMCs, and plasma was measured by qPCR using GFP primers. Shown are technical duplicates. HDAd5-GFP was included as a positive control with known erythrocyte binding via CAR.

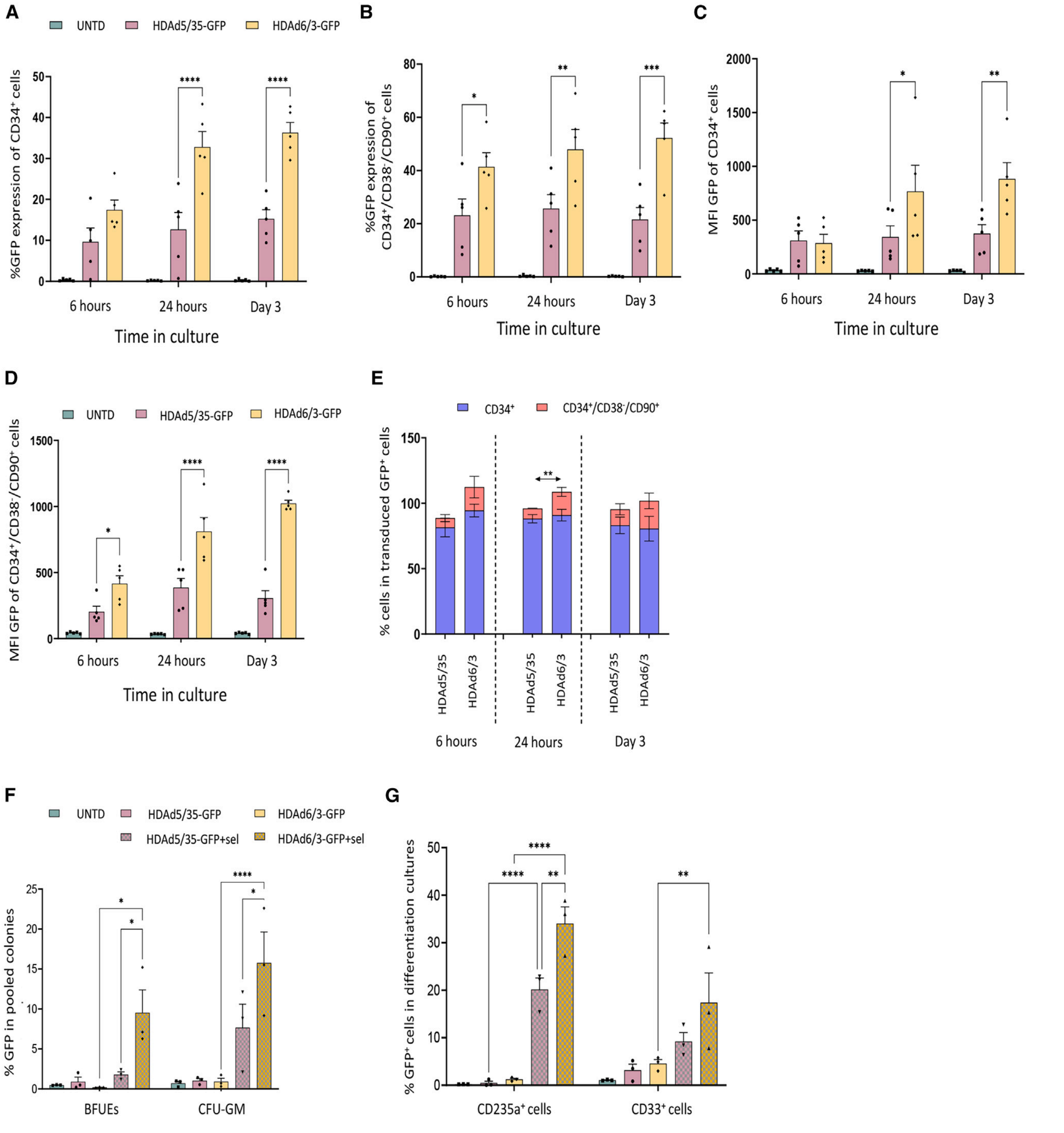

Figure 2. Higher transduction efficiency of HDAd6/3 vectors compared with HDAd5/35 vectors in human CD34$^+$ cells *in vitro*

(A and B) Percentage of GFP expression in (A) human CD34$^+$ and (B) human CD34$^+$/CD90$^+$ cells at 6 h, 24 h, and 3 days post transduction with HDAd5/35-GFP or HDAd6/3-GFP at an MOI of 4,000 vp/cell. (C and D) Mean fluorescence intensity (MFI) of GFP expression in (C) human CD34$^+$ and (D) human CD34$^+$/CD90$^+$ cells at 6 h, 24 h, and 3 days post transduction. (E) Immunophenotypic characterization of GFP$^+$ cells at multiple timepoints during culture. (F and G) Analysis of long-term transduction efficiency. Cells were transduced with HDAd5/35- or HDAd6/3-GFP vectors, along with an HDAd-SB vector for the integration of the GFP transgene. On day 7 post transduction, cells were treated with O^{6}BG/BCNU selection (HDAd5/35-GFP+sel, HDAd6/3+sel) for the *in vitro* selection of transduced cells. (F) GFP expression in pooled colonies derived from cells transduced by HDAd5/35-GFP or HDAd6/3-GFP. (G) GFP expression on CD235a$^+$ cells in erythroid differentiation and CD33$^+$ cells in myeloid differentiation culture. All plots represent data from four different donors. Data are shown as means (SD). ****$p \leq 0.0001$, ***$p \leq 0.001$, **$p \leq 0.01$, *$p \leq 0.05$ (two-way ANOVA with Bonferroni correction).

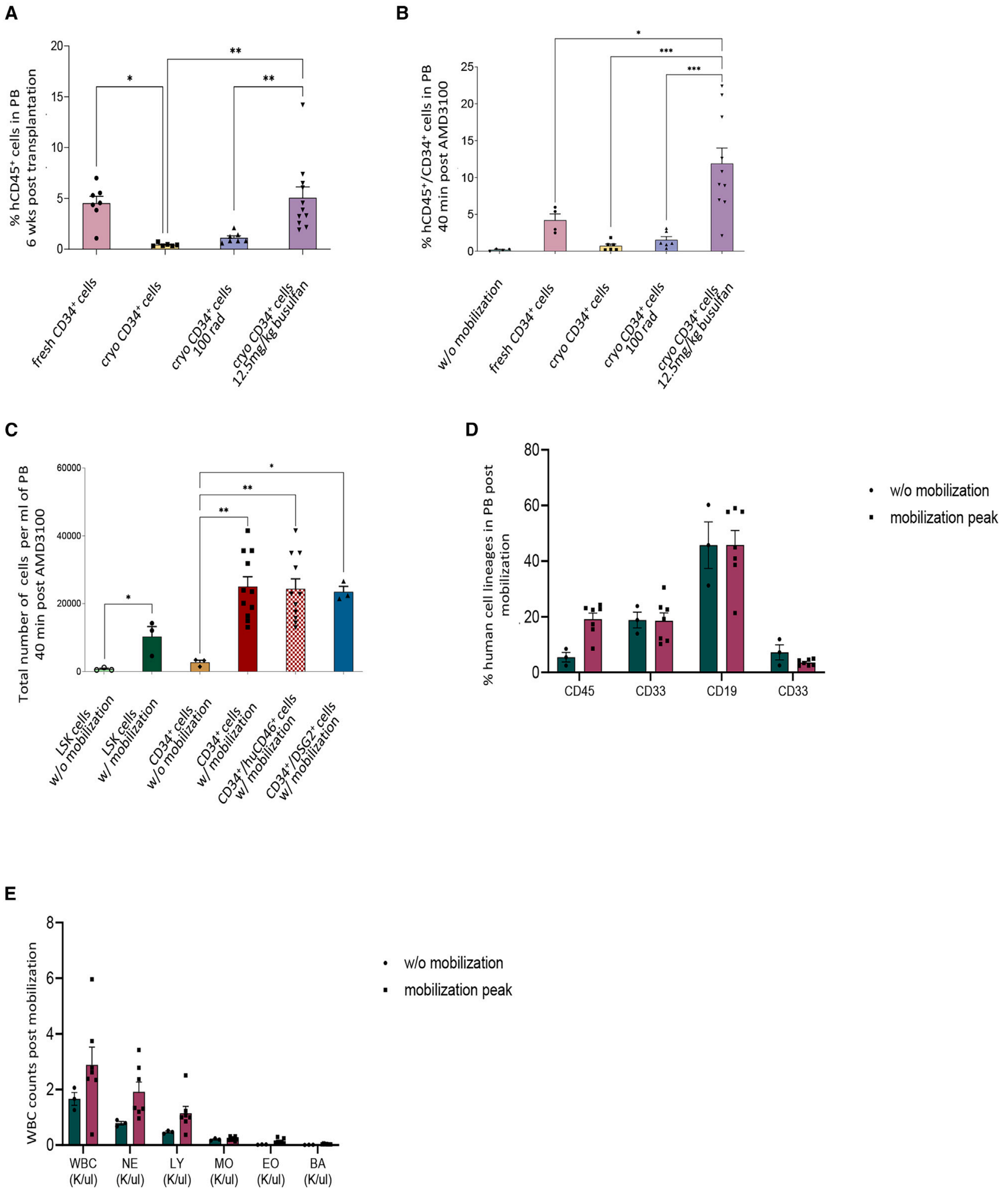

Figure 3. Busulfan conditioning enhances the engraftment of cryopreserved human CD34$^+$ cells and promotes efficient mobilization of human HSPCs

(A) Donor chimerism in peripheral blood 6 weeks post transplantation of fresh vs. cryopreserved human CD34$^+$ cells (1×10^6 cells) into NSGW41 mice with or without conditioning (100 rad TBI or busulfan 12 mg/kg). (B and C) Mobilization efficiency of G-CSF (250 μg/kg for 6 days) and AMD3100 (5 mg/kg for 4 days) assessed by (B) the

(legend continued on next page)

transduction and O^6BG/BCNU treatment, human $CD34^+$ cells were seeded in semisolid media with cytokines that allow for erythroid and myeloid differentiation. At day 12 after plating, erythroid progenitor colonies (BFU-E: burst-forming units–erythroid) and myeloid progenitors (CFU-GM: colony-forming units-granulocytes/macrophages) were pooled and analyzed for GFP. GFP expression was largely lost in progenitor colonies in settings without O^6BG/BCNU selection. In BFU-Es derived from O^6BG/BCNU-treated cells, 9.53% (2.84%) vs. 1.8% (0.35%) ($p = 0.04$) expressed GFP after transduction with HDAd6/3-GFP and HDAd5/35-GFP, respectively (Figure 2F). In CFU-GMs, 15.73% (3.88%) vs. 7.67% (2.94%) ($p = 0.0348$) cells were GFP-positive, respectively. Studies in liquid erythroid or myeloid differentiation medium confirmed the data in progenitor colonies with showing significantly higher percentage of GFP^+ cells in erythroid and myeloid cells derived from HDAd6/3-GFP transduced human $CD34^+$ cells (Figure 2F). The transduction of human $CD34^+$ cells with HDAd5/35-GFP or HDAd6/3-GFP did not affect their viability in culture (Figure S2A) nor their clonogenic ability (Figure S2B). Interestingly, HDAd6/3-GFP transduction resulted in less stress signaling, as measured by reactive oxygen species (ROS) mean fluorescence intensity (MFI), 24 h post transduction, compared with HDAd5/35-GFP (Figure S2C).

This *in vitro* study shows that the HDAd6/3 vector platform results in efficient transduction of primitive HSC subsets within human $CD34^+$ cells that are capable of multilineage expansion with an integrated transgene.

Partial myeloablation increases human chimerism and mobilization by G-CSF+AMD3100

We and others have previously reported that the *in vivo* transduction of human $CD34^+$ cells in humanized NSGW41 mice is feasible by HDAd5/35 vectors[2,28,29] after G-CSF/AMD3100 mobilization of human HSCs from the chimeric bone marrow to the periphery. These studies were performed without bone marrow preconditioning. For efficient mobilization it is important to achieve high levels of human chimerism after human $CD34^+$ cell transplantation. We therefore tested the engraftment capacity of freshly isolated and cryopreserved human $CD34^+$ cells in the NSGW41 mouse model, with or without prior partial myeloablative conditioning by either total body irradiation (TBI) or busulfan (BU). Freshly isolated human $CD34^+$ cells achieved higher engraftment rates compared with the cryopreserved $CD34^+$ cells, as reflected by the percentage of human $CD45^+$ cells in the peripheral blood, 6 weeks post transplantation (fresh human $CD34^+$: 4.53% (0.69%) vs. cryo human $CD34^+$: 0.48% (0.06%), $p = 0.0191$) (Figure 3A). However, fresh sources of human $CD34^+$ cells are not always available. For this reason, we assessed the effect of mild conditioning on the engraftment of cryopreserved human $CD34^+$ cells. Conditioning with 12.5 mg/kg BU, 48 h before transplantation of human $CD34^+$ cells (1×10^6 cells), resulted in a significantly higher percentage of human $CD45^+$ cells, in the periphery of mice 6 weeks post transplantation, when compared with mice without pre-treatment ($p = 0.0029$) or mice that received 100 rad TBI ($p = 0.0083$) (Figure 3A). Busulfan conditioning led to increased mobilization of human $CD34^+$ cells from the bone marrow to the PB by G-CSF + AMD3100, as exhibited by the highest percentage of circulating human $CD45^+/CD34^+$ cells in the PB (BU-treated: 11.89% (2.12%) vs. TBI-treated: 1.54% (0.45%), $p = 0.0004$ vs. w/o pre-treatment: 0.718% (0.26%) $p = 0.0002$ vs. fresh human $CD34^+$: 4.2% (0.86%) $p = 0.0299$), measured 40 min after the last AMD3100 injection (Figure 3B). As expected, G-CSF/AMD3100 mobilization also increased the total PB number of endogenous mouse $Lin^-/Sca1^+/cKit^+$ (LSK cells), a fraction that is enriched for HSCs (Figure 3C). The total number of mobilized HSCs was on average 28,000 human $CD34^+$ cells/mL PB (Figure 3C). All mobilized human $CD34^+$ cells were positive for CD46 and DSG2, the target receptors for HDAd5/35 and HDAd6/3 vectors, respectively (Figures 3C and S1). Based on the data from this study, for the following *in vivo* experiments, mice were transplanted with cryopreserved human $CD34^+$ cells at 48 h post BU conditioning.

In vivo HDAd transduction in humanized mice mobilized with G-CSF+AMD3100 (HDAd5/35-GFP vs. HDAd6/3-GFP)

Briefly, our *in vivo* transduction approach consists of transplantation of human $CD34^+$ cells in partially myeloablated NSGW41 mice (12.5 mg/kg busulfan) and engraftment of human HSCs (Figure 4A). Six weeks after transplantation, HSCs were mobilized with a 7-day G-CSF/AMD3100 mobilization scheme,[30] followed by an intravenous injection of the HDAd5/35-GFP + HDAd5/35-SB or HDAd6/3-GFP + HDAd6/3-SB vectors at the peak of mobilization. Four weeks post *in vivo* transduction, a group of mice received intraperitoneally O^6BG/BCNU to expand transduced HSPCs. One month later, the mice were euthanized, and the hematopoietic tissues were harvested for analysis.

At the time mice were euthanized, multilineage engraftment in the bone marrow was evident in all mice (Figure S3A). Based on flow cytometry of human $CD45^+$ cells, donor chimerism in the bone marrow was 38%–58% in all groups of mice. The *in vivo* transduction/selection resulted in similar HSC and lineage distribution of human cells ($CD34^+$, $CD33^+$, $CD19^+$, $CD3^+$, $CD235a^+$) in the bone marrow (Figure S3A) and spleen (Figure S3B) for all treatment groups, with the exception of bone marrow $CD33^+$ cells that were significantly higher in O^6BG/BCNU-treated groups. Human chimerism in the PB was in the range of 10% and stable after week 9 until the end of the experiment (Figure 4B). GFP flow cytometry of PBMCs

percentage of human $CD45^+/CD34^+$ cells and (C) the total number of human $CD45^+/CD34^+$ cells, in peripheral blood 40 min post the last injection of AMD3100. For the quantification of the total number, the percentage of the double-positive cells (human $CD45^+CD34^+$) was multiplied by the total number or the white blood cells (WBCs). (D) Analysis of human lineages in the peripheral blood at the peak of mobilization (*N* = 3 w/o mobilization, *N* = 7 mobilized mice). (E) WBC counts in non-mobilized (*N* = 3) and mobilized mice (*N* = 7). Each symbol represents an individual mouse. Data are shown as means (SD). $^{***}p \leq 0.001$, $^{**}p \leq 0.01$, $^{*}p \leq 0.05$ (two-way ANOVA with Bonferroni correction).

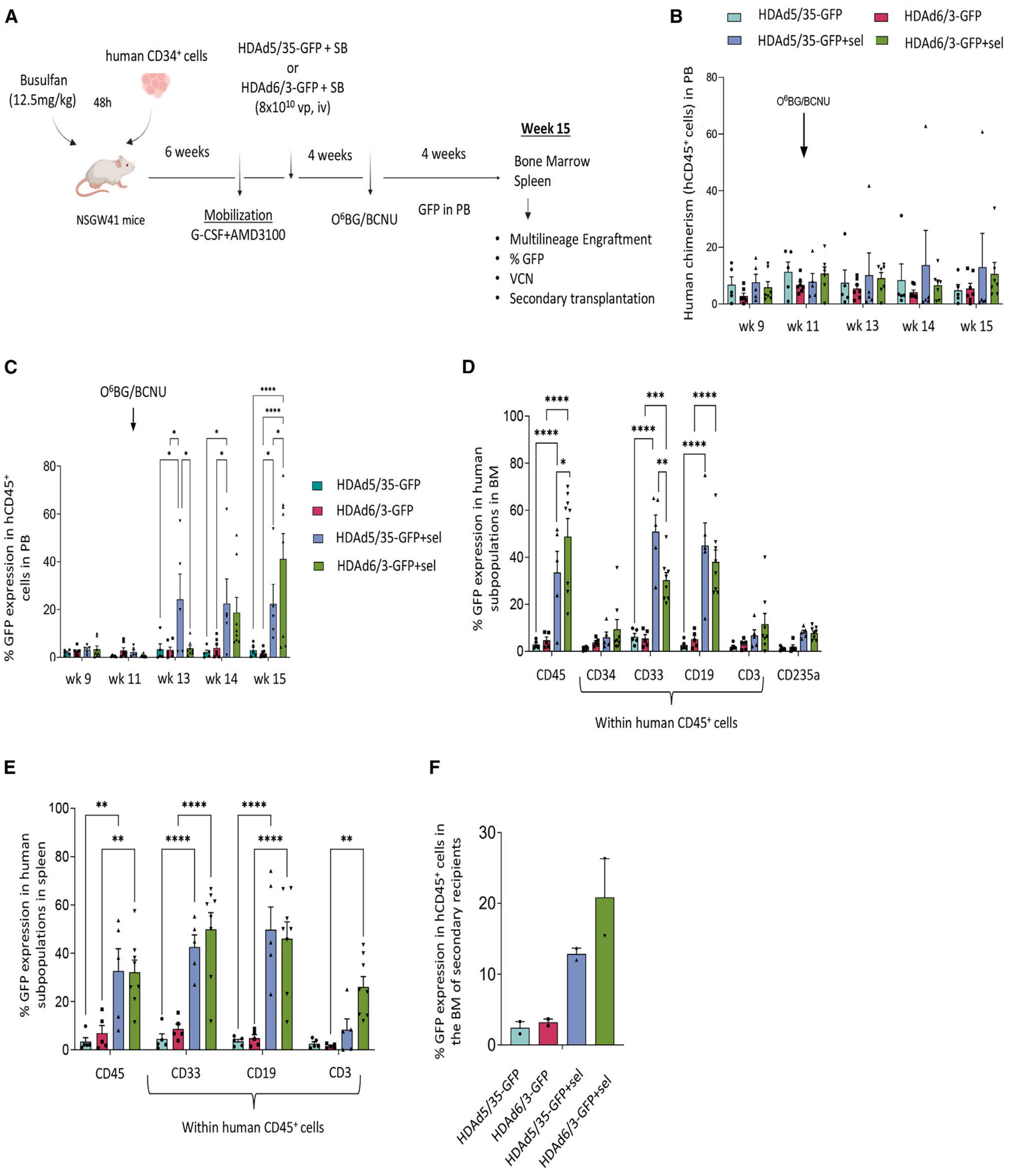

Figure 4. Efficient *in vivo* transduction of human HSPCs using the HDAd6/3-GFP vector platform

(A) Overview of the experimental design of *in vivo* transduction experiments. Briefly, human $CD34^+$ cells from healthy donors were transplanted into busulfan-treated NSGW41 mice ($N = 23$; HDAd5/35-GFP: $N = 5$, HDAd6/3-GFP: $N = 5$, HDAd5/35-GFP+sel: $N = 5$, and HDAd6/3-GFP+sel: $N = 8$). Six weeks post transplantation, HSCs were mobilized from the bone marrow to the peripheral blood by G-CSF and AMD3100. At the peak of mobilization, mice were injected intravenously with HDAd5/35-GFP or HDAd6/3-GFP vectors along with their respective Sleeping Beauty-expressing HDAd vectors. One month post *in vivo* transduction, a subset of the mice underwent a single

(legend continued on next page)

showed that *in vivo* selection with O6BG/BCNU led to increased GFP expression of human $CD45^+$ cells in the PB transduced by HDAd5/35-GFP or HDAd6/3-GFP, compared with their untreated counterparts (Figure 4C). One month post *in vivo* selection (week 15), the time of euthanization, HDAd6/3-treated mice showed significantly higher percentages of human $CD45^+/GFP^+$ cells compared with HDAd5/35-treated mice (41.12% [10.62%] vs. 22.38% [8.17%], p = 0.0338, respectively) (Figure 4C). This was also reflected by the percentage of GFP-expressing human $CD45^+$ cells in the bone marrow (Figure 4D) and spleen (Figure 4E), which reached 53.51% (6.95%) and 33.75% (5.47%), respectively, in HDAd6/3-GFP-treated/*in vivo* selected animals. The major subfractions that contributed to high marking rates in these groups were myeloid CD33 and lymphoid (CD19, CD3) cells (Figures 4D and 4E). O6BG/BCNU treatment also increased the percentage of $GFP^+/CD235a^+$ erythroid cells in the bone marrow, a subfraction that, overall, is relatively small in humanized mice due to poor erythroid differentiation (Figure 4D). At the time of mice were euthanized (week 15), isolated human $CD45^+$ cells from the bone marrow of the *in vivo* transduced mice were isolated and transplanted into individual BU-preconditioned secondary NSGW41 recipients (1 donor:1 recipient). Nine weeks post transplantation, the secondary recipients were euthanized, and their bone marrow was analyzed. It showed increased GFP expression in the human cells, a pattern similar to the one from the primary mice (Figure 4F).

Taken together, the outcome of these studies indicates that the HDAd6/3 vector platform can efficiently transduce human HSPCs *in vivo*, resulting in 41.12% (10.62%) and 53.51% (6.95%) of GFP marking in PBMCs and bone marrow mononuclear cells, respectively.

In vivo HSC transduction with a penton-modified HDAd5/35_lam vector in humanized mice after G-CSF/AMD3100 mobilization

Next, we tested another new vector platform, HDAd5/35_lam. Atasheva et al. modified first-generation Ad5/35 vectors on multiple levels including the fiber, hexon, and penton.[31] We tried to reproduce their penton modification in our HDAd5/35-GFP vector by replacing the RGD motif with the SIKVAV motif derived from laminin chain A. This should redirect the virion from $\alpha_v\beta_{3/5}$ integrins to a different internalization receptor, $\alpha_6\beta_1$ (CD49f). This receptor is expressed in primitive HSCs[1] and a corresponding penton-modified HDAd vector (HDAd5/35_lam-GFP) should more efficiently transduce these cells than the parental HDAd5/35-GFP vector. We tested this in humanized mice that were mobilized with G-CSF/AMD3100 and intravenously injected with HDAd5/35-GFP + HDAd-SB or HDAd5/35_lam-GFP + HDAd-SB, followed by one round of O6BG/BCNU selection. One cohort of mice was euthanized on day 3 to assess transduction before *in vivo* selection (Figure 5A). On day 3, all mice exhibited similar levels of multilineage reconstitution of bone marrow (Figure S4A) and spleen (Figure S4B). There were no significant differences in transduction of human $CD45^+$ cells in the PB based on GFP expression (Figures 5B and 5C). However, in the bone marrow, transduction of human $CD34^+$ cells and more primitive human $CD34^+/CD38^-$ and human $CD34^+/CD38^-/CD45RA^-$ cells was more efficient for HDAd5/35_lam-GFP-injected mice (Figures 5D–5F). In long-term studies, *in vivo* selection expanded HDAd5/35_GFP transduced HSCs and, with a delayed effect, HDAd5/35_lam-GFP transduced human $CD45^+$ cells at week 15 in the PB, without significant differences between the two vectors (HDAd5/35-GFP: 25.95 (9.49) vs. HDAd5/35_lam-GFP: 32.15 (4.79), p = 0.9821) (Figure 5G). In the bone marrow, at the end of the study (week 15), GFP marking in human $CD34^+$ cells and more primitive subsets, like human $CD34^+/CD38^-/CD90^+/CD45RA^+$, was significantly higher in HDAd5/35_lam-GFP transduced mice (Figure 5H, 5I, and S5). However, in week 15, higher GFP marking rates with HDAd5/35_lam in primitive HSCs did not translate into higher marking rates in the PB human $CD45^+$ cells (Figure 5G) or human cells in the spleen (Figure S6). We speculate that the HSC subfraction(s) that HDAd5/35_lam transduces cannot efficiently differentiate or exit the bone marrow in the time frame of 15 weeks.

G-CSF-free HSPC mobilization in humanized mice

As a mobilizing agent, G-CSF has a number of disadvantages. It requires multiple doses, is known to alter the function of the HSC niche, as well as bone formation, and mobilizes variable numbers of HSCs. A critical problem associated with G-CSF is unselective mobilization of committed cells leading to sequestration of vectors and high peripheral cytokine levels released from granulocytes that interacted with HDAd. Leukocytosis and release of pro-inflammatory cytokines from granulocytes would be particularly critical in sickle cell disease patients.[32] We therefore tested G-CSF-free mobilization regimens involving the small synthetic molecules tGroβ (aka MGTA-145), a cxcr2-inhibitor, and WU-106, a VLA4 inhibitor. In CD46 transgenic mice we showed previously that these regimens more rapidly, efficiently, and selectively mobilize mouse HSCs.[33,34] Here we tested these methods in humanized mice. We did a side-by-side comparison of the mobilization kinetics of (1) G-CSF mixed with AMD3100, (2) tGroβ mixed with AMD3100, and (3) all three reagents together (tGroβ+WU-106+AMD3100). The rationale of the triple combination was because they use different mechanisms for mobilization and should act additively. All reagents were subcutaneously injected together once and the percentages of human $CD45^+/CD34^+$ cells (Figure 6A) and more primitive human

round of *in vivo* selection by O6BG/BCNU. One month later, the mice were euthanized, and their hematopoietic tissues were harvested for further analysis. (B) Human chimerism in peripheral blood. (C) GFP expression of human $CD45^+$ cells in peripheral blood. (D) GFP expression of human subpopulations in BM. (E) GFP expression of human subpopulations in spleen. (F) At week 15, the *in vivo* transduced mice were euthanized and human $CD45^+$ cells were isolated from the chimeric bone marrow; the isolated cells were subsequently individually transplanted into busulfan-treated NSGW41 mice (secondary recipients) (one donor to one recipient) (N = 8; HDAd5/35-GFP: N = 2, HDAd6/3-GFP: N = 2, HDAd5/35-GFP+sel: N = 2 and HDAd6/3-GFP+sel: N = 2). Nine weeks post transplantation, the secondary recipients were euthanized, and bone marrow cells were analyzed for GFP expression in the human $CD45^+$ cells. Each symbol represents an individual mouse. Data are shown as means (SD). $^{****}p \leq 0.0001$, $^{***}p \leq 0.001$, $^{**}p \leq 0.01$, $^{*}p \leq 0.05$ (two-way ANOVA with Bonferroni correction).

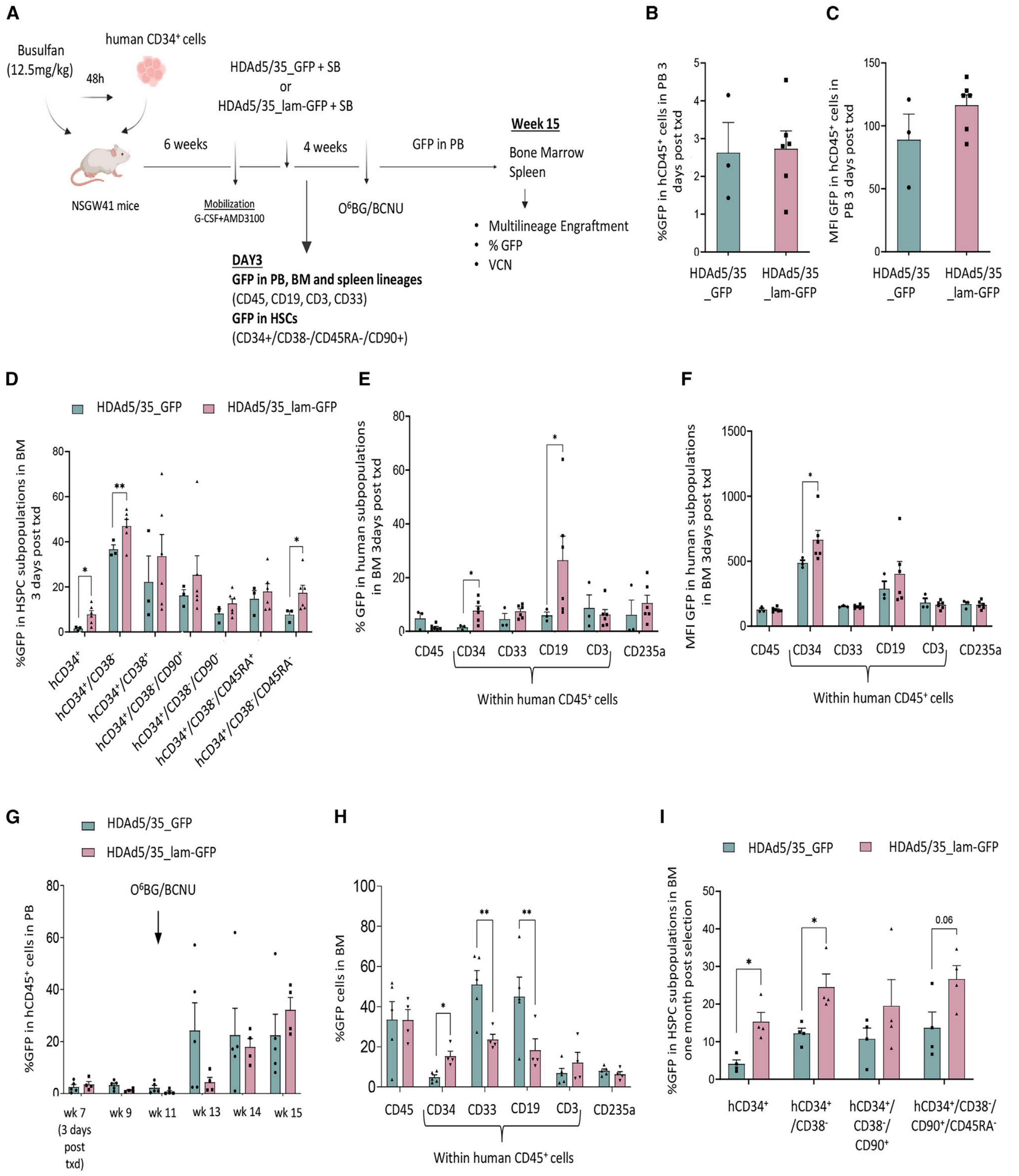

Figure 5. Enhanced *in vivo* transduction efficiency of human HSPCs using the HDAd5/35 laminin vector platform

(A) Overview of experimental design. The new HDAd5/35 laminin vector targets two HSC receptors: the HSC attachment receptor (CD46) and the HSC internalization receptor ($\alpha_6/\beta_{1/4}$ integrins - CD49f). CD49f expression is predominantly expressed on primitive HSCs. Retargeting to this receptor was achieved by replacing the RGD (Arg-Gly-Asp) motif within the Ad5 penton with the functional core motif of laminin chain A IKVAV (Ile-Lys-Val-Ala-Val). Humanized mice were mobilized with G-CSF/AMD3100 and

(legend continued on next page)

CD45$^+$/CD34$^+$/CD90$^+$ cells (Figure 6B) in PB were measured at 15 min; 1, 2, 3, and 6 h; and 3 days after injection. The peak of G-CSF+AMD3100 mobilization was at ~1 h. The peaks for tGroβ+AMD and tGroβ+WU106+AMD3100 were at 15 min post injection. Importantly, mobilization of human CD34$^+$/CD90$^+$ with tGroβ+AMD3100 was significantly more efficient than G-CSF+AMD3100 at their corresponding peaks (Figure 6B). Along this line, the best mobilization of primitive human CD34$^+$/CD90$^+$ was achieved with the triple combination (Figure 6B). Human HSPCs were still detectable in the peripheral blood circulation on day 3 indicating that their return to bone marrow and spleen could be a limiting factor for *in vivo* HSC transduction. The different mobilization regimens did not change the lineage distribution at week 15 in spleen and bone marrow regardless of *in vivo* selection (Figures S7A–S7C). In addition to measuring HSC mobilization based on surface markers, we also conducted a functional progenitor CFU assay. This assay did not show differences between G-CSF+AMD3100 and tGroβ+AMD3100 mobilization, and the triple combination of mobilization drugs was even less efficient (Figure 6C). This is in line with the finding that the addition of WU-106 to tGroβ+AMD3100 reduced the mobilization of CD34$^+$ cells (Figure 6A) and CFU-GM (Figure 6E). However, it increased the mobilization of primitive CD34$^+$ subfractions such as CD34$^+$/CD90$^+$ cells, indicating that WU-106 acts on more primitive cells. The conclusion from this study is that tGroβ+AMD3100 and tGroβ+WU-106+AMD3100 result in efficient and rapid mobilization of primitive human CD34$^+$/CD90$^+$ HSCs.

In vivo HSC transduction after mobilization of humanized mice with different drugs

HDAd6/3-GFP was injected into humanized mice at the peak of the respective mobilization regimen (Figure 7A). Four weeks later, mice received one round of treatment with O^{6}BG/BCNU and were then followed until week 15. At this time point, human chimerism in bone marrow based on the percentage of human CD45$^+$ cells was 39.55% (4.64%), 63.83% (7.96%), and 29.86% (6.96%) for G-CSF+AMD3100, tGroβ+AMD3100, and tGroβ+WU106+AMD3100 mobilized animals (Figure 7B). The percentage of human CD33$^+$ cells in the bone marrow was significantly higher for the G-CSF+AMD3100 mobilization regimen (Figure 7B). However, within spleen (Figure S7A) no significant differences in lineage distribution was seen for the three mobilization regimens. GFP expression was measured in PB human CD45$^+$ cells from week 7 (3 days post HDAd) to week 15 (Figure 7C). There was a peak at day 3 post HDAd that most likely originated from direct transduction of circulating human cells by HDAd6/3-GFP. This GFP expression was then lost due to turnover of transduced cells. O^{6}BG/BCNU treatment resulted in a steady increase in GFP marking rates, which reached, at week 15, 41.12% (10.63%), 11.68% (2.61%), and 8.48% (2.54%) of GFP$^+$/human CD45$^+$ cells after mobilization with G-CSF+AMD3100, tGroβ+AMD3100, and tGroβ+WU-106+AMD3100. Similar GFP expression pattern was observed in human CD45$^+$ cells within the bone marrow, where the percentage of GFP was 48.8% (7.67%), 17.1% (6.92%), and 21.1% (8.67%), respectively (Figure 7D). However, this was not translated to the rest of the cell lineages in bone marrow (Figure 7D). GFP marking rates were about 10-fold lower in animals that did not receive O^{6}BG/BCNU selections (Figures S7D–S7E). Quantification of the transgene copy number in bone marrow human CD34$^+$ cells did not show any statistically significant differences among mice mobilized with the three different mobilization schemes (Figure 7F). Noteworthy is the GFP marking in HSPCs of the spleen at week 15. Of human CD34$^+$ cells, 22.41% (3.32%), 39.7% (9.32%), and 34.88% (6.17%) were GFP-positive, respectively, for the three mobilization groups (Figure 7E). This was not primarily due to preferential engraftment of human CD34$^+$ cells after transplantation or due to preferential return of mobilized human CD34$^+$ to the spleen (Figure S7G). The high percentage of GFP-expressing human CD34$^+$ cells at the end of the study indicates that transduced mobilized human CD34$^+$ cells expanded after O^{6}BG/BCNU treatment. Without selection, GFP expression in splenic human CD34$^+$ cells was ~1% (Figure S7F). We also speculate that transduced HSCs in the spleen contributed to GFP-positive PBMCs after O^{6}BG/BCNU.

DISCUSSION

NSGW41 mice engraft cryopreserved human CD34$^+$ cells in the bone marrow without preconditioning; however, human chimerism in peripheral blood is usually low, and mobilization of human cells by G-CSF+AMD3100 for *in vivo* HSC transduction is inefficient. We show that low-dose total body irradiation (100 Rad) or busulfan treatment (12.5 mg/kg) increases the percentage of human CD45$^+$ cells in PBMCs 2- and 5-fold, respectively. This implies that after transplantation of cryopreserved human CD34$^+$ cells, the relatively low human/mouse chimerism in peripheral blood can be overcome by partial myelo-conditioning. In low-dose busulfan-conditioned humanized mice, 6 weeks after human CD34$^+$ cell transplantation, G-CSF/AMD3100 mobilization resulted in >20,000 human CD34$^+$ cells in the periphery at 40 min after AMD3100 injection (15% of human CD45$^+$ cells were CD34$^+$ positive). Because G-CSF/AMD3100 mobilization is problematic, specifically in patients with sickle cell disease, we used G-CSF-free mobilization

in vivo transduced by HDAd3/35_GFP or HDAd3/35_lam-GFP. A subset of mice was euthanized 3 days post *in vivo* transduction, to assess the early transduction efficiency. One month post *in vivo* transduction the remaining mice received a single dose of O^{6}BG/BCNU selection drugs. Four weeks post *in vivo* selection, the mice were euthanized, and their hematopoietic tissues were harvested for further analysis. (B–F) Early transduction efficiency, 3 days post *in vivo* transduction (txd). (B) Percentage of GFP expression in the human CD45$^+$ cells in the peripheral blood (PB). (C) Mean fluorescence intensity (MFI) of GFP expression in the human CD45$^+$ cells in the PB. (D) Early *in vivo* transduction efficiency measured by %GFP in human HSPCs in the BM, 3 days post virus injection. (E) GFP expression of human subpopulations in bone marrow on day 3 post *in vivo* txd. (F) MFI of GFP in human cells in chimeric bone marrow on day 3 of *in vivo* transduction. (G–I) Long-term *in vivo* transduction efficiency of human HSPCs. (G) Percentage of GFP-expressing human CD45$^+$ cells in peripheral blood. (H) Percentage of GFP expression in human subpopulations in bone marrow. (I) Percentage of GFP expression in human HSPCs in the bone marrow of humanized mice 1 month post *in vivo* transduced/selection. Each symbol represents an individual mouse. Data are shown as means (SD). $^{**}p \leq 0.01$, $^{*}p \leq 0.05$ (two-way ANOVA with Bonferroni correction).

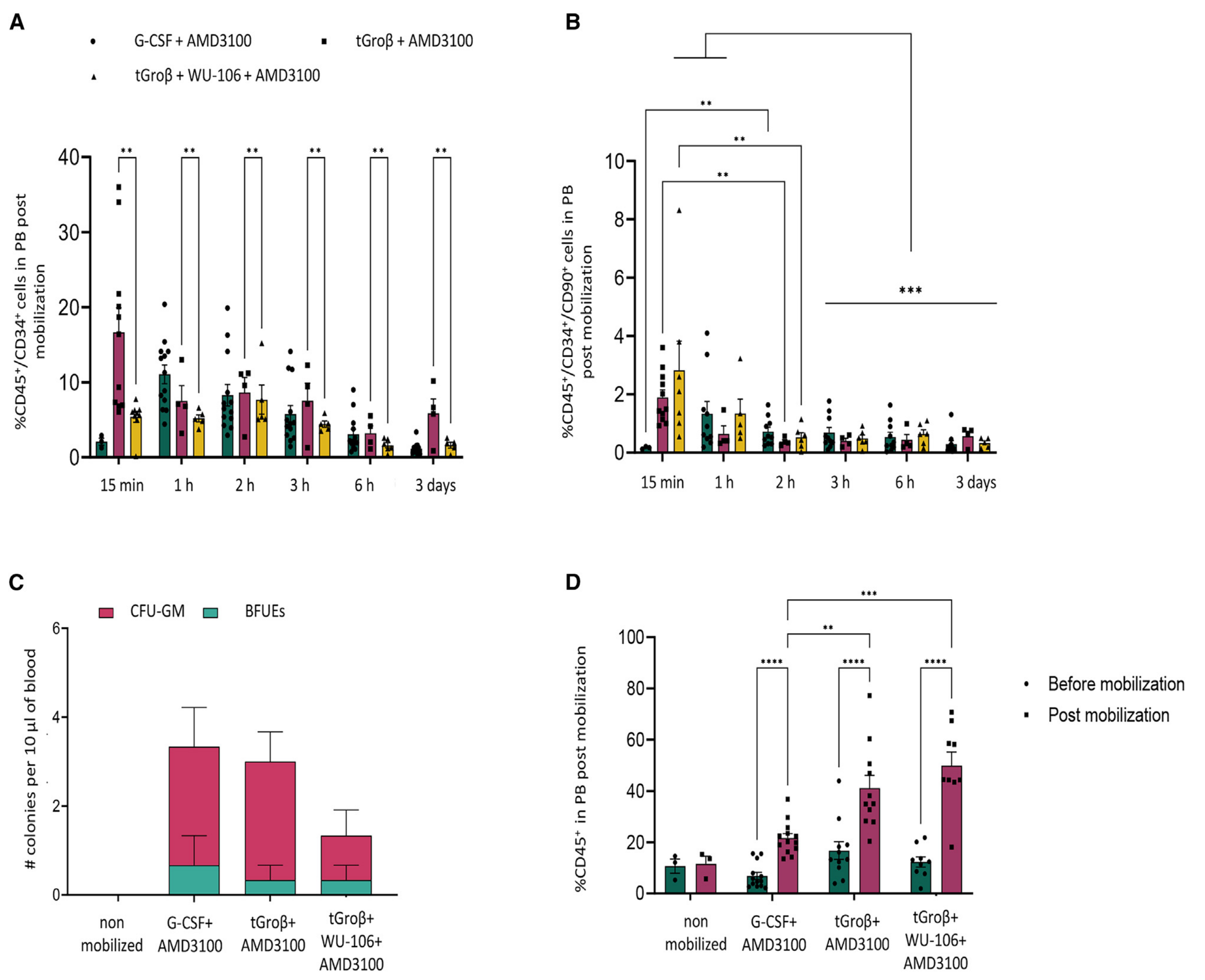

Figure 6. Efficient mobilization of human CD34$^+$ cells in humanized NSGW41 mice using G-CSF-free mobilization approaches

Three different mobilization strategies were employed to mobilize human HSPCs from the bone marrow into the peripheral blood: (1) G-CSF (250 µg/kg for 6 days) and AMD3100 (5 mg/kg for 4 days), (2) tGroβ (2.5 mg/kg) and AMD3100 (5 mg/kg), administered as a single injection, and (3) WU-106 (10 mg/kg) and AMD3100 (5 mg/kg) as a single injection followed by administration of tGroβ (2.5 mg/kg) and AMD3100 (5 mg/kg), 1.5 h later. (A and B) Mobilization kinetics was evaluated at multiple timepoints, assessed as (A) the percentage of human CD45$^+$/CD34$^+$ cells and (B) the percentage of human CD45$^+$/CD34$^+$/CD90$^+$ cells. (C) The number of CFUs was analyzed from 10 µL peripheral blood collected at the peak of mobilization either 40 min post last AMD3100 injection or 15 min post tGroβ injection. (D) The percentage of human CD45$^+$ cells at the peak of mobilization for each scheme. Each symbol represents an individual mouse. Data are shown as means (SD). ****$p \leq 0.0001$ ***$p \leq 0.001$, **$p \leq 0.01$ (two-way ANOVA with Bonferroni correction). The G-CSF+AMD3100 group transduced with the HDAd6/3 vector is the same as in Figure 4.

regimens, the CXCR2-inhibitor tGroβ together with AMD3100 or AMD3100-tGroβ together with the VLA4 inhibitor WU-106. Choo et al. reported that robust mobilization of phenotypic HSCs out of the bone marrow (BM) using G-CSF+AMD3100 could be achieved in NBSGW mice; however, most mobilized HSCs were not detected in the PB.[35] This discrepancy with our observation could be because we used a different G-CSF+AMD3100 mobilization regimen (6 days of G-CSF and 4 days of AMD3100) and new reagents (tGroβ and WU-106) that use different pathways for mobilization.

At the peak of mobilization, humanized mice were intravenously injected with a CD46-targeting HDAd5/35-GFP vector or a DSG2-targeting HDAd6/3-GFP vector. The corresponding receptors are present on 100% of mobilized human HSCs. Independently, Choo et al. demonstrated that CD46 and DSG2 were abundantly expressed on the surface of the human CD34$^+$ cells extracted from humanized mice.[35] Expression of DSG2 and CD46 on HSCs was also confirmed by Yao et al., whereby DSG2 was preferentially expressed in a subset of CD34$^+$ cells that was enriched for primitive HSCs,[1] which are the ultimate target cells of our *in vivo* HSC gene therapy approach. We

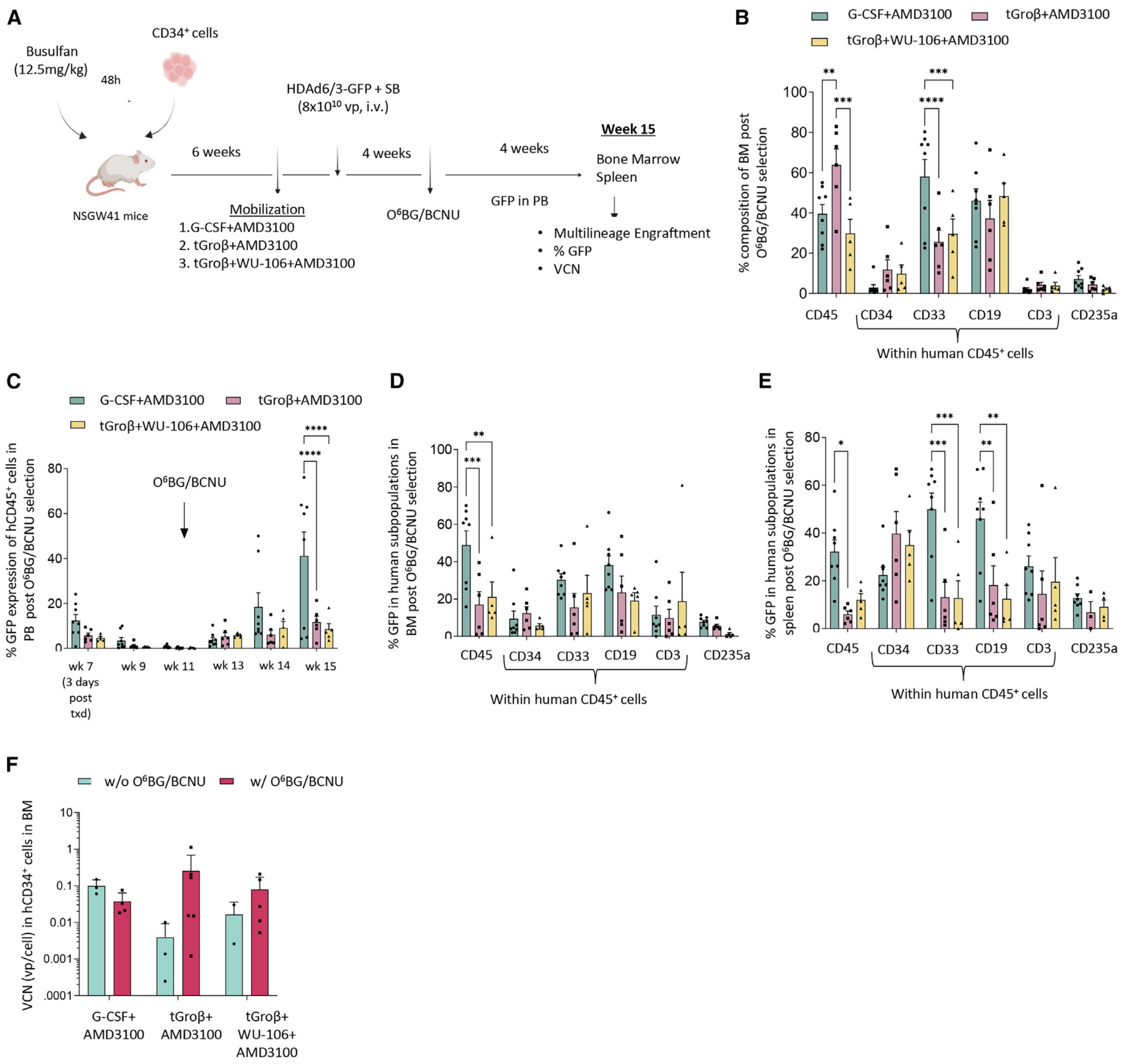

Figure 7. G-CSF-free mobilization approaches yield HSPCs that are effectively transduced *in vivo* by HDAd6/3-GFP vectors

(A) Overview of experimental design. Briefly, human $CD34^+$ cells from healthy donors were transplanted into busulfan-treated NSGW41 mice (*N* = 19; G-SCF+AMD3100: *N* = 8, tGroβ+AMD3100: *N* = 6 and WU-106+tGroβ+AMD3100: *N* = 5). Six weeks post transplantation, HSCs were mobilized from the bone marrow to the peripheral blood by three different mobilization approaches. At the peak of each mobilization approach, mice were *in vivo* transduced by HDAd6/3-GFP and HDAd6/3-SB vector platform, followed by a single round of *in vivo* selection by O^6BG/BCNU 1 month later. Four weeks post *in vivo* selection, the mice were euthanized, and their hematopoietic tissues were harvested for further analysis. (B) Multilineage composition of human cells in bone marrow (BM). (C–E) Percentage of GFP-expressing cells in the hematopoietic tissues of mice post *in vivo* selection. (C) In human $CD45^+$ cells in the peripheral blood, (D) in human subpopulations in BM, and (E) in human subpopulations in spleen. (F) Vector Copy Number (VCN) in human $CD34^+$ cells isolated from the bone marrow of HDAd6/3-*in vivo* transduced mice with or without *in vivo* selection. Each symbol represents an individual mouse. Data are shown as means (SD). $^{****}p \leq 0.0001$, $^{***}p \leq 0.001$, $^{**}p \leq 0.01$, $^{*}p \leq 0.05$ (two-way ANOVA with Bonferroni correction).

therefore introduced in our studies the new HDAd6/3+ vector platform. *In vitro* studies confirmed greater transduction of primitive HSCs, i.e., $CD34^+/CD38^-/CD90^+$ cells and colony-forming cells by HDAd6/3-GFP compared with HDAd5/35-GFP. This was also confirmed in *in vivo* studies in humanized mice where we also showed that *in vivo* transduced/selected $CD34^+$ cells give rise to GFP^+ cells in all lineage-positive cells. Stable GFP marking levels in peripheral blood human $CD45^+$ cells were ∼40%. At end of the study (week

15), in G-CSF+AMD3100-mobilized animals, ~10% and ~25% of GFP^+CD34^+ cells were found in the bone marrow and spleen, respectively (Figures 7D and 7E). A similar trend was seen in animals with tGroβ+AMD3100 and tGroβ+WU-106+AMD3100 mobilization. From data shown in Figure S7G, we concluded that the high percentage of transduced $CD34^+$ cells in the spleen is due to O^6BG/BCNU expansion or preferential return of mobilized and *in vivo* transduced HSCs return to the spleen. This could also explain the relatively low percentage of $GFP^+/CD34^+$ cells in bone shown in Figure 5D. This finding is in agreement with our studies in rhesus macaques where we found the highest vector copy number per cell in the spleen.[3,22] We speculate that a similar situation will be found in human studies. Our studies in NHPs also indicate that the ratio of transgene-expressing HSCs in bone marrow and spleen can be reversed by expression of CXCR4.[3]

We also tried to improve the HDAd5/35 vector platform by targeting the virus to a second HSC receptor, $\alpha_6\beta_1$/CD49f. The idea of targeting Ad vectors to an alternative integrin was first tested by the Shayakhmetov group.[31] Later, Yao et al. also showed efficient *in vivo* HSC transduction in humanized mice with a hexon-, penton-, and fiber-modified vector derived from Ad5.[1] Single intravenous administration of this vector into G-CSF+AMD3100-mobilized mice led to up to 20% transduction of human $CD34^+CD38^-CD45RA^-$ HSPC subsets in the bone marrow. In HDAd5/35_lam, we replaced the $\alpha_v\beta_{3/5}$ binding RGD motif with a motif derived from laminin (IKVAV). We found >20% transduction of human $CD34^+CD38^-CD90^+$ 3 days after HDAd injection, which is in agreement with the findings by Yao et al. While they did not analyze long-term GFP marking in peripheral human $CD45^+$ cells, we found no differences in this parameter between HDAd5/35-GFP vs. HDAd5/3_lam-GFP. We speculate that we did not wait long enough for the transduced HSCs to differentiate and exit the bone marrow. It is also notable that the vector used by Yao et al. contained other capsid modifications, in addition to the RGD motif substitution.

Our data with different mobilization drugs indicate that tGroβ+-AMD3100 (also in combination with WU-106) is superior in mobilizing more primitive HSCs that can then be transduced *in vivo*. Selective mobilization of HSCs would reduce vector sequestration by committed cells, thereby increasing the transduction of primitive HSC. It would also decrease the efflux of activated granulocytes from the bone marrow.

While *in vivo* HSC transduction studies in humanized mice allow for conclusions about the *in vivo* tropism HDAds toward HSCs after intravenous injection, this model has also major limitations. NSGW41 mice, due to the combined immunodeficiency background (reflected by macrocytic anemia, thrombocytosis, and lymphopenia) tend to be morbid and have a relatively short lifespan. Human chimerism is restricted to the hematopoietic system, which makes meaningful biodistribution and toxicity studies in non-hematopoietic tissues with new *in vivo* delivery vehicles impossible. Human hematopoietic progenitors, specifically erythroid and lymphoid progenitors, do not terminally differentiate in this model.[36,37] This complicates studies aimed toward correcting genetic defects in erythrocytes and lymphocytes. In addition, xenotransplantation of mice as adults significantly shortens the study duration because animals are usually 8 to 12 weeks old at the time of transplantation and assessment of human chimerism is not feasible until animals are 4–5 months old. Development of human T cells, although slow and inefficient, can lead to graft vs. host disease. Potentially, the latter problems can be circumvented by intrahepatic transplantation of human $CD34^+$ cells into neonates. On the other hand, this technique is technically more complex.

In summary, our data in humanized mice show that human HSCs can be efficiently mobilized with G-CSF/AMD3100 or G-CSF-free regimens and *in vivo* transduced with HDAd6/3 vectors. One cycle of chemo-selection results in stable GFP marking rates in PBMCs of 40%, which, theoretically, should be curative for thalassemia and sickle cell disease.

MATERIALS AND METHODS

Reagents

Before transplantation of human $CD34^+$ cells, busulfan (Mylan Institutional LLC) was administrated to NSGW41 mice. For mobilization and *in vivo* transduction, G-CSF (Neupogen) (Amgen, Thousand Oaks, CA), AMD3100 (MilliporeSigma, Burlington, MA), and dexamethasone sodium phosphate (Fresenius Kabi USA, Lake Zurich, IL) were used. tGroβ was provided by Ensoma, Inc (Boston, MA). WU-106 has been described previously.[33]

Helper dependent adenovirus vectors (HDAds)

HDAd5/35 contained the Ad5 capsid with the fiber proteins substituted with those of Ad35. The affinity of the Ad35 fiber to CD46 was enhanced by mutations.[38] HDAd6/3 is derived from serotype Ad6,[2] with the Ad6 fiber knob replaced with an Ad3 fiber knob. The affinity of the Ad3 fiber knob to DSG2 was increased by mutations.[24] To construct the Ad6/3 helper virus, the plasmid pAd6/35k++[2] was digested with AsiSI, the ~8.8-kb fragment containing Ad6 tail, shaft and Ad35 knob (Ad35k++) was then replaced by another ~8.8-kb new fragment containing Ad6 tail, shaft, and Ad3 knob (Ad3k+) using Gibson assembly, the new fragment was generated by gene synthesis (Genscript). To generate Ad5/35_lam helper vector, we have replaced the RGD (Arg-Gly-Asp) motif within the Ad5 penton with the functional core motif of laminin chain A SIKVAV (Ser-Ile-Lys-Val-Ala-Val) to generate Ad5/35_lam as described by Dmitry Shayakhmetov's group.[1] All HDAd vectors have the same transgene cassette consisting of a ubiquitously active EF1a promoter driving a $mgmt^{P140}$/GFP cassette.[16] HDAd-SB contains expression cassettes for SB100x and Flpe and is used to mediate integration of the mgtm/GFP cassette.[16] For the production of HDAd vectors, corresponding plasmids were linearized with *PmeI* and rescued in 116 cells[39] with Ad5/35++ helper virus[40] (for HDAd5/35 vectors), Ad6/3 helper virus[2] (for HDAd6/3 vectors), or Ad5/35_lam helper virus (for HDAd5/35_lam). HDAd vectors were produced in 116 cells as described in detail elsewhere.[39] Helper virus

contamination levels were found to be <0.05%. Titers were 3–9 × 10^{12} vp/mL.

SDS-polyacrylamide gel electrophoresis analysis of viral proteins

Viral proteins were reduced in 0.25 M Tris-Cl (pH 6.8)–8% SDS–1% β-mercaptoethanol–10% glycerol with boiling for 5 min and then loaded on a Tris-glycine-SDS–10% polyacrylamide gel, run at 35 mA for 3 h, and silver-stained as described previously.[41]

Dynamic light scattering

Ad particles were diluted to 1 × 10^{10} vp/mL in a 100-μL PBS buffer before running samples on a Malvern Zetasizer Nano-S Series (Malvern Panalytical, Malvern, United Kingdom).

RBC binding assays

Human blood was used for the assessment of blood cell binding of HDAd5/35, HDAd5/35_lam, and HDAd6/3 particles. HDAd5 was used as positive control, as it is known to bind to human erythrocytes.[26] Whole heparinized blood (5 × 10^9 erythrocytes/mL) was incubated with 2 × 10^8/mL copies of the HDAds for 30 min with constant shaking. After that, the blood was centrifuged (1,000 × *g* for 5 min) and the plasma, erythrocytes, and PBMCs were collected for further analysis. The erythrocytes and PBMCs were washed three times with PBS. DNA was extracted using the DNeasy Blood and Tissue kit (Qiagen) according to the manufacturer's instructions. Real-time quantitative PCR was performed by sampling 5 μL of the extracted DNA in duplicate in a 10 μL reaction in StepOne Plus (Applied Biosystems), using the PowerSYBR Green PCR Master Mix (Applied Biosystems) and GFP primers. DNA from a GFP plasmid was serially diluted and served as a standard curve.

Human $CD34^+$ cell culture

Fresh and frozen aliquots of $CD34^+$ cells from mobilized healthy donors were obtained from the Fred Hutchinson Cancer Center, Seattle, Washington. The cells were recovered from frozen stocks and incubated for 24 h in serum-free medium (Stemspan SFEMII, Stemcell Technologies) supplemented with penicillin/streptomycin (Gibco), Flt3 ligand (Flt3-L, 100 ng/mL), thrombopoietin (TPO, 100 ng/mL), and Stem Cell Factor (SCF, 100 ng/mL) and the small molecules StemRegenin1 (SR1, 1 μM) (Cellagen Technology) and Ly2228820 (Ly, 100 nM) (Selleckchem). All cytokines were obtained from PeproTech.

$CD34^+$ cells were transduced with the HDAd5/35-GFP or HDAd6/3-GFP vectors along with the HDAd-SB vector (for integration) in low-attachment plates for 48 h, at a total MOI of 4,000 vp/cell, before transferred in erythroid differentiation-, myeloid differentiation-, and methylcellulose-based medium. Seven days after transduction, cells were treated once with 50 μM O^6BG and 35 μM BCNU for *in vitro* selection of cells with integrated vector copies.

In vitro erythroid differentiation of human $CD34^+$ cells

Differentiation of transduced and non-transduced human $CD34^+$ cells into erythroid cells was done based on a 3-step protocol developed by Douay et al.[42] In step 1, cells were cultured at a density of 10^4 cells/mL for 7 days in a basal medium containing Iscove's modified Dulbecco's medium (IMDM), 5% human plasma, glutamine, Pen-Strep, heparin (2 IU/mL), insulin (10 μg/mL), Holo-Transferrin (330 μg/mL) supplemented with hydrocortisone (1 μM), SCF (100 ng/mL), interleukin (IL)-3 (5 ng/mL), and erythropoietin (EPO) (3 U/mL). In step 2, cells were cultured at a density of 10^5 cells/mL for 4 days in the same basal medium, as previously, supplemented with SCF (100 ng/mL) and EPO (3 U/mL). Finally, in step 3, the cells were cultured at a density of 10^6 cells/mL for 7–10 additional days in the same medium, supplemented only with EPO (3 U/mL).

In vitro myeloid differentiation of human $CD34^+$ cells

Differentiation of human $CD34^+$ cells into myeloid cells was done based on the following protocol: 10^4 cells/mL were cultured in medium containing IMDM, 5% human plasma, glutamine, Pen-Strep, heparin (2 IU/mL), supplemented with SCF (50 ng/mL), IL-3 (5 ng/mL), granulocyte-macrophage colony-stimulating factor (GM-CSF) (10 ng/mL), and G-CSF (20 ng/mL), for 15–18 days. All cytokines were from PeproTech.

Animal studies

All experiments involving animals were conducted in accordance with the institutional guidelines set forth by the University of Washington. The University of Washington receives accreditation from the Association for the Assessment and Accreditation of Laboratory Animal Care International (AALAC) and all live animal work conducted at the university is in accordance with the Office of Laboratory Animal Welfare (OLAW) Public Health Assurance (PHS) policy, USDA Animal Welfare Act and Regulations, the Guide for the Care and Use of Laboratory Animals, and the University of Washington's Institutional Animal Care and Use Committee (IACUC) policies. The studies were approved by the University of Washington IACUC (Protocol No. 3108-01).

Mobilization and *in vivo* transduction of human $CD34^+$ cells in a humanized NSGW41 mouse model

The immunodeficient NOD.Cg-Kit W-41J Prkdc scid Il2rg tm1Wjl/WaskJ (NSGW41) mice were generously provided by Thalia Papayannopoulou (University of Washington). A humanized model was generated by transplanting human $CD34^+$ cells from healthy donors into NSGW41 mice (1 × 10^6/recipient), post partial myeloablation (busulfan 12.5 mg/kg). Six weeks post transplantation, the mice, having a human bone marrow chimerism, were mobilized by a 7-day mobilization scheme, including G-CSF 250 μg/kg subcutaneously (days 1–6) and AMD3100 5 mg/kg intraperitoneally (i.p.) (days 5–7), as previously described.[30] Forty minutes after the last AMD3100 injection, mice received intravenously (i.v.) the HDAd5/35-GFP + HDAd-SB or HDAd6/3 + HDAd-SB or HDAd5/35_Lam-GFP + HDAd-SB vectors at a total dose of 8 × 10^{10} viral particles (divided into two doses, 30 min apart). Sixteen and 2 h before i.v. injection of HDAd vectors, the animals received dexamethasone (i.p, 10 mg/kg). One month post *in vivo* transduction, the animals were

injected i.p. with freshly prepared O^6BG (30 mg/kg, in two doses, 30 min apart) and 5 mg/kg BCNU for the *in vivo* selection of transduced cells. One month post *in vivo* selection, NSGW41 mice were euthanized, and their hematopoietic tissues were collected, for assessment of multilineage engraftment and transduction efficiency. Human $CD45^+$ cells were isolated from the bone marrow and transplanted into secondary NSGW41 recipients. A small fraction of these cells was used for CFU cultures. Notably, immunodeficient NOD.Cg-Kit W-41J Prkdc scid Il2rg tm1Wjl/WaskJ (NSGW41) mice have several mutations that affect DNA repair mechanisms and are therefore more sensitive to DNA damaging agents such as O^6BG/BCNU. Unlike *in vivo* selection in CD46 transgenic mice, which is performed with 3–4 increasing doses of BCNU, NSGW41 mice tolerate only one O^6BG/low-dose BCNU treatment.

Colony-forming unit (CFU) cultures

A total of 2,000–3,000 human $CD34^+$ cells, or 5×10^4 human $CD45^+$ cells isolated from chimeric bone marrow were plated in semisolid methylcellulose-based medium containing cytokines, MethoCult H4434 (StemCell Technologies), according to the manufacturer's instructions. After 11–14 days of incubation, CFUs were classified and enumerated under a light microscope by trained operators. Suspensions of pooled colonies were made with at least 25 CFU-GM or BFUE colonies for the analysis of GFP expression.

Cell surface staining/flow cytometry

To evaluate the HSC phenotype post transduction, cells were washed and stained with the following fluorochrome-conjugated antibodies against human antigens: CD34-APC, CD38-PE, CD90-PerCP, and CD45RA-APC-H7 (BD Biosciences, San Jose, CA). For the follow-up of human $CD34^+$ cell differentiation into erythroid or myeloid cells the following antibodies were used: For erythroid cells: CD235a-PE (BD Biosciences) and CD71 FITC (BD Biosciences); for myeloid cells: CD33-PE (BD Biosciences). To assess the multilineage engraftment of human $CD34^+$ cells in the bone marrow of NSGW41 mice post transplantation, the following antibodies were used: CD45-APC (BD Biosciences), CD19-PerCP (BioLegend), CD3-FITC (BioLegend), CD33-PE (BD Biosciences), and CD235a-PE (BD Biosciences). To measure the percentage of human HSPCs in peripheral blood of NSGW41 mice post mobilization, the following antibodies were used: CD45-APC (BD Biosciences, San Jose, CA), CD34-PE (BioLegend), and CD90-PerCP (BD Biosciences, San Jose, CA). Blood was collected retro-orbitally at various time points post mobilization and stained with the antibodies for 15 min in a cool and dark place. After the incubation, erythroid cells were lysed using an RBC Lysis Buffer (BioLegend), followed by a washing step in FACS buffer. After wash, cells were resuspended in FACS buffer and analyzed using a BD FACSymphony A3 Cell Analyzer (BD Biosciences, San Jose, CA). Debris was excluded using a forward scatter-area and sideward scatter-area gate. Flow cytometry data were then analyzed using FlowJo (version10.0.8, FlowJo, LLC). A complete list of antibodies can be found in Table S1. Notably, it is not uncommon that the percentages of CD33, CD19, and CD3 do not sum to 100% of human CD45. Several factors can contribute to this. Primarily, gating is often focused on $CD33^{high}$ expressing cells. However, it is known that some subsets of monocytes and macrophages, even though they express CD45, may downregulate the CD33 expression. In addition, NSGW41 mice support, to some extent, the differentiation to dendritic cells and natural killer (NK) cells, which express CD45 (but not CD33, CD3, or CD19). Last, because of the mouse model limitations, a significant number of immature cells are present that express the pan-leucocyte marker CD45, but lack strong expression of CD33, CD19, and CD3.

Magnetic cell sorting

Human $CD45^+$ and/or $CD34^+$ cells from chimeric bone marrow were isolated using human CD45 or CD34 Microbeads (cat# 130-045-801, cat#130-046-702, respectively) (Miltenyi Biotec, San Diego, CA) according to the manufacturer's instructions. The positive fractions were used for the secondary transplantation assays and the CFU cultures. The human $CD34^+$ cells were for the analysis of vector copy number (VCN).

VCN analysis

Total DNA of human $CD45^+$ or human $CD34^+$ cells isolated from bone marrow, spleen, and peripheral blood was extracted by DNeasy Blood and Tissue kit (Qiagen) according to the manufacturer's instructions. Real-time quantitative PCR was performed with 9.6 ng DNA in duplicate in a 10-μL reaction in StepOne Plus (Applied Biosystems), using the PowerSYBR Green PCR Master Mix (Applied Biosystems) and the following primers: GFP F: 5′-TCGTGACCAC CCTGACCTAC-3′, GFP R: 5′-GGTCTTGTAGTTGCCGTCGT-3′, huGAPDH: 5′-CAAATTCCATGGCACCGTCA-3′, huGAPDH F: 5′-TCCTAGTTGCCTCCCCAAAG-3′. DNA from GFP was serially diluted and served as a standard curve. Thermal cycling started for 10 min at 95°C followed by 40 thermal cycles of 15 s at 95°C and 1 min at 60°C. Human GAPDH was used as a control gene. Transgene-specific signals were normalized to GAPDH (taken as two copies per cell, which allowed the calculation of the cell number in the given DNA preparation).

Statistics

Data are presented as mean (SD). For comparisons of multiple groups, two-way ANOVA with Bonferroni post hoc testing was employed. Student t test was used for comparisons between two groups. Statistical analysis was performed using GraphPad Prism version 10.0.3 (GraphPad Software Inc.). p values less than 0.05 were considered significant.

DATA AVAILABILITY

All raw data used in this study will be made available upon request.

ACKNOWLEDGMENTS

The study was supported by NIH grants R01HL128288 (A.L.) and R01HL141781 (A.L.), by a grant from Ensoma Bio (A.L.), and by a grant from the Bill and Melinda Gates Foundation (BMGF): INV-017692 (A.L.). Under the grant conditions of the BMGF, a Creative Commons Attribution 4.0 Generic License has already been assigned to the Author Accepted Manuscript version that might arise from this submission. We thank Theo Koob, Lishan Huang, and Anna Anderson for technical assistance.

AUTHOR CONTRIBUTIONS

A.L. provided the conceptual framework for the study. A.G. and A.L. designed the experiments. A.G. performed the experiments. H.W. and C.L. provided critical material and advice. A.G. and A.L. wrote the manuscript.

DECLARATION OF INTERESTS

A.L. is an academic co-founder of Ensoma Bio without payment.

SUPPLEMENTAL INFORMATION

Supplemental information can be found online at https://doi.org/10.1016/j.omtm.2025.101448.

REFERENCES

1. Yao, J., Atasheva, S., Wagner, N., Di Paolo, N.C., Stewart, P.L., and Shayakhmetov, D.M. (2024). Targeted, safe, and efficient gene delivery to human hematopoietic stem and progenitor cells in vivo using the engineered AVID adenovirus vector platform. Mol. Ther. *32*, 103–123. https://doi.org/10.1016/j.ymthe.2023.10.023.
2. Wang, H., Georgakopoulou, A., Zhang, W., Kim, J., Gil, S., Ehrhardt, A., and Lieber, A. (2023). HDAd6/35++ - A new helper-dependent adenovirus vector platform for in vivo transduction of hematopoietic stem cells. Mol. Ther. Methods Clin. Dev. *29*, 213–226. https://doi.org/10.1016/j.omtm.2023.03.008.
3. Wang, H., Germond, A., Li, C., Gil, S., Kim, J., Kiem, H.P., and Lieber, A. (2022). In vivo HSC transduction in rhesus macaques with an HDAd5/3+ vector targeting desmoglein 2 and transiently overexpressing cxcr4. Blood Adv. *6*, 4360–4372. https://doi.org/10.1182/bloodadvances.2022007975.
4. Wang, H., Georgakopoulou, A., Li, C., Liu, Z., Gil, S., Bashyam, A., Yannaki, E., Anagnostopoulos, A., Pande, A., Izsvák, Z., et al. (2020). Curative in vivo hematopoietic stem cell gene therapy of murine thalassemia using large regulatory elements. JCI Insight *5*, e139538.
5. Li, C., Wang, H., Georgakopoulou, A., Gil, S., Yannaki, E., and Lieber, A. (2021). In Vivo HSC Gene Therapy Using a Bi-modular HDAd5/35++ Vector Cures Sickle Cell Disease in a Mouse Model. Mol. Ther. *29*, 822–837. https://doi.org/10.1016/j.ymthe.2020.09.001.
6. Li, C., Georgakopoulou, A., Mishra, A., Gil, S., Hawkins, R.D., Yannaki, E., and Lieber, A. (2021). In vivo HSPC gene therapy with base editors allows for efficient reactivation of fetal gamma-globin in beta-YAC mice. Blood Adv. *5*, 1122–1135. https://doi.org/10.1182/bloodadvances.2020003702.
7. Li, C., Psatha, N., Sova, P., Gil, S., Wang, H., Kim, J., Kulkarni, C., Valensisi, C., Hawkins, R.D., Stamatoyannopoulos, G., and Lieber, A. (2018). Reactivation of gamma-globin in adult beta-YAC mice after ex vivo and in vivo hematopoietic stem cell genome editing. Blood *131*, 2915–2928. https://doi.org/10.1182/blood-2018-03-838540.
8. Wang, H., Georgakopoulou, A., Psatha, N., Li, C., Capsali, C., Samal, H.B., Anagnostopoulos, A., Ehrhardt, A., Izsvák, Z., Papayannopoulou, T., et al. (2019). In vivo hematopoietic stem cell gene therapy ameliorates murine thalassemia intermedia. J. Clin. Investig. *129*, 598–615. https://doi.org/10.1172/JCI122836.
9. Li, C., Mishra, A.S., Gil, S., Wang, M., Georgakopoulou, A., Papayannopoulou, T., Hawkins, R.D., and Lieber, A. (2019). Targeted Integration and High-Level Transgene Expression in AAVS1 Transgenic Mice after In Vivo HSC Transduction with HDAd5/35++ Vectors. Mol. Ther. *27*, 2195–2212. https://doi.org/10.1016/j.ymthe.2019.08.006.
10. Li, C., Psatha, N., Wang, H., Singh, m., Samal, H.B., Zhang, W., Ehrhardt, A., Izsvák, Z., Papayannopoulou, T., and Lieber, A. (2018). Integrating HDAd5/35++ vectors as a new platform of HSC gene therapy of hemoglobinopathies. Mol. Ther. Methods Clin. Dev. *9*, 142–152. https://doi.org/10.1016/j.omtm.2018.02.004.
11. Wang, H., Richter, M., Psatha, N., Li, C., Kim, J., Liu, J., Ehrhardt, A., Nilsson, S.K., Cao, B., Palmer, D., et al. (2018). A Combined In Vivo HSC Transduction/Selection Approach Results in Efficient and Stable Gene Expression in Peripheral Blood Cells in Mice. Mol. Ther. Methods Clin. Dev. *8*, 52–64. https://doi.org/10.1016/j.omtm.2017.11.004.
12. Neff, T., Horn, P.A., Peterson, L.J., Thomasson, B.M., Thompson, J., Williams, D.A., Schmidt, M., Georges, G.E., von Kalle, C., and Kiem, H.P. (2003). Methylguanine methyltransferase-mediated in vivo selection and chemoprotection of allogeneic stem cells in a large-animal model. J. Clin. Investig. *112*, 1581–1588. https://doi.org/10.1172/JCI18782.
13. Beard, B.C., Trobridge, G.D., Ironside, C., McCune, J.S., Adair, J.E., and Kiem, H.P. (2010). Efficient and stable MGMT-mediated selection of long-term repopulating stem cells in nonhuman primates. J. Clin. Investig. *120*, 2345–2354. https://doi.org/10.1172/JCI40767.
14. Kim, J., Li, C., Wang, H., Kaviraj, S., Singh, S., Savergave, L., Raghuwanshi, A., Gil, S., Germond, A., Baldessari, A., et al. (2022). Translational development of a tumor junction opening technology. Sci. Rep. *12*, 7753. https://doi.org/10.1038/s41598-022-11843-z.
15. Kemper, C., Leung, M., Stephensen, C.B., Pinkert, C.A., Liszewski, M.K., Cattaneo, R., and Atkinson, J.P. (2001). Membrane cofactor protein (MCP; CD46) expression in transgenic mice. Clin. Exp. Immunol. *124*, 180–189.
16. Richter, M., Saydaminova, K., Yumul, R., Krishnan, R., Liu, J., Nagy, E.E., Singh, M., Izsvák, Z., Cattaneo, R., Uckert, W., et al. (2016). In vivo transduction of primitive mobilized hematopoietic stem cells after intravenous injection of integrating adenovirus vectors. Blood *128*, 2206–2217. https://doi.org/10.1182/blood-2016-04-711580.
17. Li, C., Georgakopoulou, A., Newby, G.A., Chen, P.J., Everette, K.A., Paschoudi, K., Vlachaki, E., Gil, S., Anderson, A.K., Koob, T., et al. (2023). In vivo HSC prime editing rescues sickle cell disease in a mouse model. Blood *141*, 2085–2099. https://doi.org/10.1182/blood.2022018252.
18. Wang, H., Liu, Z., Li, C., Gil, S., Papayannopoulou, T., Doering, C.B., and Lieber, A. (2019). High-level protein production in erythroid cells derived from in vivo transduced hematopoietic stem cells. Blood Adv. *3*, 2883–2894. https://doi.org/10.1182/bloodadvances.2019000706.
19. Li, C., Course, M.M., McNeish, I.A., Drescher, C.W., Valdmanis, P.N., and Lieber, A. (2020). Prophylactic in vivo hematopoietic stem cell gene therapy with an immune checkpoint inhibitor reverses tumor growth in syngeneic mouse tumor models. Cancer Res. *80*, 549–560. https://doi.org/10.1158/0008-5472.CAN-19-1044.
20. Wang, H., Beyer, I., Persson, J., Song, H., Li, Z., Richter, M., Cao, H., van Rensburg, R., Yao, X., Hudkins, K., et al. (2012). A new human DSG2-transgenic mouse model for studying the tropism and pathology of human adenoviruses. J. Virol. *86*, 6286–6302. https://doi.org/10.1128/JVI.00205-12.
21. Wang, H., Li, Z.Y., Liu, Y., Persson, J., Beyer, I., Möller, T., Koyuncu, D., Drescher, M.R., Strauss, R., Zhang, X.B., et al. (2011). Desmoglein 2 is a receptor for adenovirus serotypes 3, 7, 11 and 14. Nat. Med. *17*, 96–104. https://doi.org/10.1038/nm.2270.
22. Li, C., Wang, H., Gil, S., Germond, A., Fountain, C., Baldessari, A., Kim, J., Liu, Z., Georgakopoulou, A., Radtke, S., et al. (2022). Safe and efficient in vivo hematopoietic stem cell transduction in nonhuman primates using HDAd5/35++ vectors. Mol. Ther. Methods Clin. Dev. *24*, 127–141. https://doi.org/10.1016/j.omtm.2021.12.003.
23. Cosgun, K.N., Rahmig, S., Mende, N., Reinke, S., Hauber, I., Schäfer, C., Petzold, A., Weisbach, H., Heidkamp, G., Purbojo, A., et al. (2014). Kit regulates HSC engraftment across the human-mouse species barrier. Cell Stem Cell *15*, 227–238. https://doi.org/10.1016/j.stem.2014.06.001.
24. Wang, H., Yumul, R., Cao, H., Ran, L., Fan, X., Richter, M., Epstein, F., Gralow, J., Zubieta, C., Fender, P., and Lieber, A. (2013). Structural and functional studies on the interaction of adenovirus fiber knobs and desmoglein 2. J. Virol. *87*, 11346–11362. https://doi.org/10.1128/JVI.01825-13.
25. Richter, M., Yumul, R., Wang, H., Saydaminova, K., Ho, M., May, D., Baldessari, A., Gough, M., Drescher, C., Urban, N., et al. (2015). Preclinical safety and efficacy studies with an affinity-enhanced epithelial junction opener and PEGylated liposomal doxorubicin. Mol. Ther. Methods Clin. Dev. *2*, 15005. https://doi.org/10.1038/mtm.2015.5.
26. Carlisle, R.C., Di, Y., Cerny, A.M., Sonnen, A.F.P., Sim, R.B., Green, N.K., Subr, V., Ulbrich, K., Gilbert, R.J.C., Fisher, K.D., et al. (2009). Human erythrocytes bind and inactivate type 5 adenovirus by presenting Coxsackie virus-adenovirus receptor and complement receptor 1. Blood *113*, 1909–1918. https://doi.org/10.1182/blood-2008-09-178459.
27. Hsu, E.C., Dörig, R.E., Sarangi, F., Marcil, A., Iorio, C., and Richardson, C.D. (1997). Artificial mutations and natural variations in the CD46 molecules from human and monkey cells define regions important for measles virus binding. J. Virol. *71*, 6144–6154.

28. Georgakopoulou, A., Li, C., Kiem, H.P., and Lieber, A. (2024). In vitro and in vivo expansion of CD33/HBG promoter-edited HSPCs with Mylotarg. Mol. Ther. Methods Clin. Dev. *32*, 101343. https://doi.org/10.1016/j.omtm.2024.101343.

29. Psatha, N., Georgakopoulou, A., Li, C., Nandakumar, V., Georgolopoulos, G., Acosta, R., Paschoudi, K., Nelson, J., Chee, D., Athanasiadou, A., et al. (2021). Enhanced HbF reactivation by multiplex mutagenesis of thalassemic CD34+ cells in vitro and in vivo. Blood *138*, 1540–1553. https://doi.org/10.1182/blood.2020010020.

30. Psatha, N., Sgouramali, E., Gkountis, A., Siametis, A., Baliakas, P., Constantinou, V., Athanasiou, E., Arsenakis, M., Anagnostopoulos, A., Papayannopoulou, T., et al. (2014). Superior long-term repopulating capacity of G-CSF+plerixafor-mobilized blood: implications for stem cell gene therapy by studies in the Hbb(th-3) mouse model. Hum. Gene Ther. Methods *25*, 317–327. https://doi.org/10.1089/hgtb.2014.005.

31. Atasheva, S., Emerson, C.C., Yao, J., Young, C., Stewart, P.L., and Shayakhmetov, D.M. (2020). Systemic cancer therapy with engineered adenovirus that evades innate immunity. Sci. Transl. Med. *12*, eabc6659. https://doi.org/10.1126/scitranslmed.abc6659.

32. Fitzhugh, C.D., Hsieh, M.M., Bolan, C.D., Saenz, C., and Tisdale, J.F. (2009). Granulocyte colony-stimulating factor (G-CSF) administration in individuals with sickle cell disease: time for a moratorium? Cytotherapy *11*, 464–471. https://doi.org/10.1080/14653240902849788.

33. Li, C., Anderson, A.K., Ruminski, P., Rettig, M., Karpova, D., Kiem, H.P., DiPersio, J.F., and Lieber, A. (2024). A simplified G-CSF-free procedure allows for in vivo HSC gene therapy of sickle cell disease in a mouse model. Blood Adv. *8*, 4089–4101. https://doi.org/10.1182/bloodadvances.2024012757.

34. Li, C., Goncalves, K.A., Raskó, T., Pande, A., Gil, S., Liu, Z., Izsvák, Z., Papayannopoulou, T., Davis, J.C., Kiem, H.P., and Lieber, A. (2021). Single-dose MGTA-145/plerixafor leads to efficient mobilization and in vivo transduction of HSCs with thalassemia correction in mice. Blood Adv. *5*, 1239–1249. https://doi.org/10.1182/bloodadvances.2020003714.

35. Choo, S., Wolf, C.B., Mack, H.M., Egan, M.J., Kiem, H.P., and Radtke, S. (2024). Choosing the right mouse model: comparison of humanized NSG and NBSGW mice for in vivo HSC gene therapy. Blood Adv. *8*, 916–926. https://doi.org/10.1182/bloodadvances.2023011371.

36. Rahmig, S., Kronstein-Wiedemann, R., Fohgrub, J., Kronstein, N., Nevmerzhitskaya, A., Bornhäuser, M., Gassmann, M., Platz, A., Ordemann, R., Tonn, T., and Waskow, C. (2016). Improved Human Erythropoiesis and Platelet Formation in Humanized NSGW41 Mice. Stem Cell Rep. *7*, 591–601. https://doi.org/10.1016/j.stemcr.2016.08.005.

37. Kyoizumi, S., Murray, L.J., and Namikawa, R. (1993). Preclinical analysis of cytokine therapy in the SCID-hu mouse. Blood *81*, 1479–1488.

38. Wang, H., Liu, Y., Li, Z., Tuve, S., Stone, D., Kalyushniy, O., Shayakhmetov, D., Verlinde, C.L.M., Stehle, T., McVey, J., et al. (2008). In vitro and in vivo properties of adenovirus vectors with increased affinity to CD46. J. Virol. *82*, 10567–10579. JVI.01308-08 [pii]. https://doi.org/10.1128/JVI.01308-08.

39. Palmer, D., and Ng, P. (2003). Improved system for helper-dependent adenoviral vector production. Mol. Ther. *8*, 846–852. S1525001603002855 [pii].

40. Palmer, D.J., and Ng, P. (2004). Physical and infectious titers of helper-dependent adenoviral vectors: a method of direct comparison to the adenovirus reference material. Mol. Ther. *10*, 792–798. [pii]. https://doi.org/10.1016/j.ymthe.2004.06.1013 S1525-0016(04)01308-5.

41. Lieber, A., He, C.Y., Kirillova, I., and Kay, M.A. (1996). Recombinant adenoviruses with large deletions generated by Cre-mediated excision exhibit different biological properties compared with first-generation vectors in vitro and in vivo. J. Virol. *70*, 8944–8960. https://doi.org/10.1128/JVI.70.12.8944-8960.1996.

42. Neildez-Nguyen, T.M.A., Wajcman, H., Marden, M.C., Bensidhoum, M., Moncollin, V., Giarratana, M.C., Kobari, L., Thierry, D., and Douay, L. (2002). Human erythroid cells produced ex vivo at large scale differentiate into red blood cells in vivo. Nat. Biotechnol. *20*, 467–472. https://doi.org/10.1038/nbt0502-467.

OMTM, Volume *33*

Supplemental information

In vivo HSC transduction in humanized mice mediated by novel capsid-modified HDAd vectors

Aphrodite Georgakopoulou, Hongjie Wang, Jiho Kim, Chang Li, and André Lieber

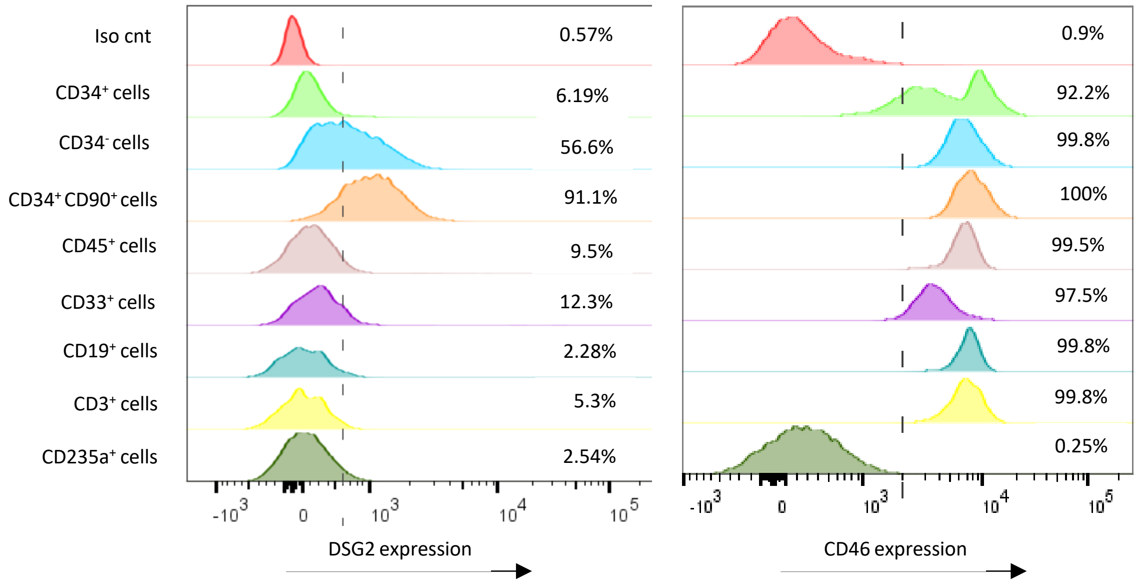

Fig.S1. Expression of HDAd attachment receptors CD46 and DSG2. Representative histograms of DSG2 and CD46 expression in human HSPCs, one day post thawing, in $CD34^-$ cell subset and several human lineages. All plots represent data from 4 different donors. Data are shown as means ± SEM. *$p \leq 0.05$ (One-way ANOVA with Bonferroni correction).

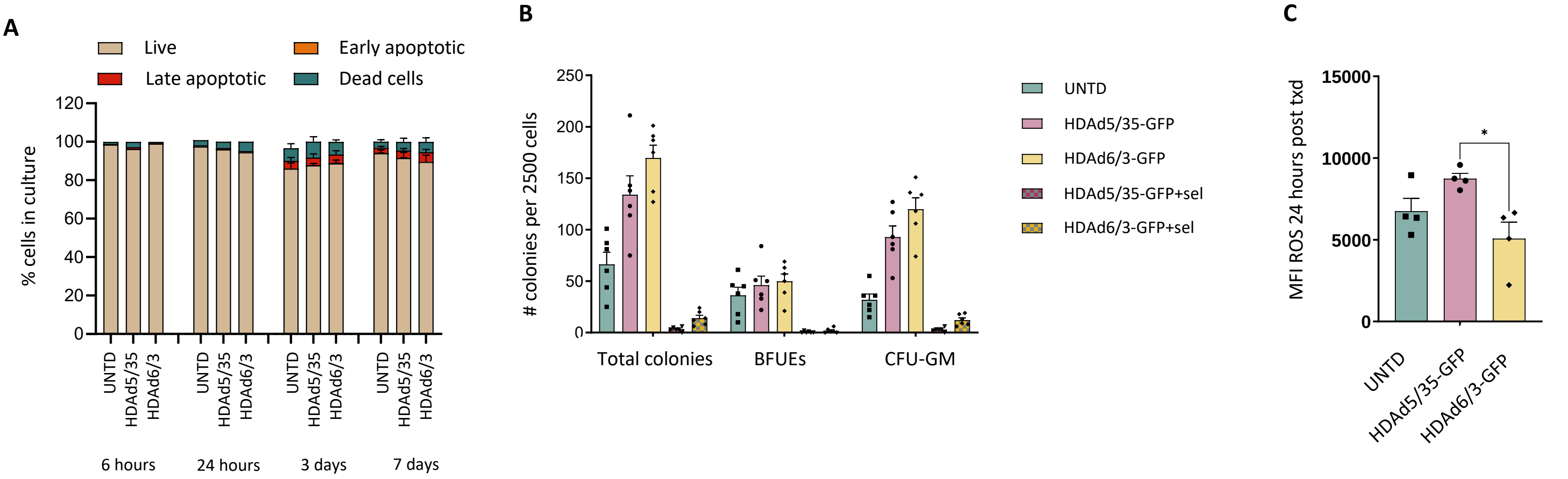

Fig.S2. Evaluation of HDAd6/3_GFP transduction efficiency in human CD34⁺ cells. A) Viability of human CD34⁺ cells 6h-, 24h-, 3 days- and 7 days- post transduction. **B)** Total colony number per 2500 human CD34⁺ cells (plated in duplicates). **C)** ROS expression 24 hours post transduction

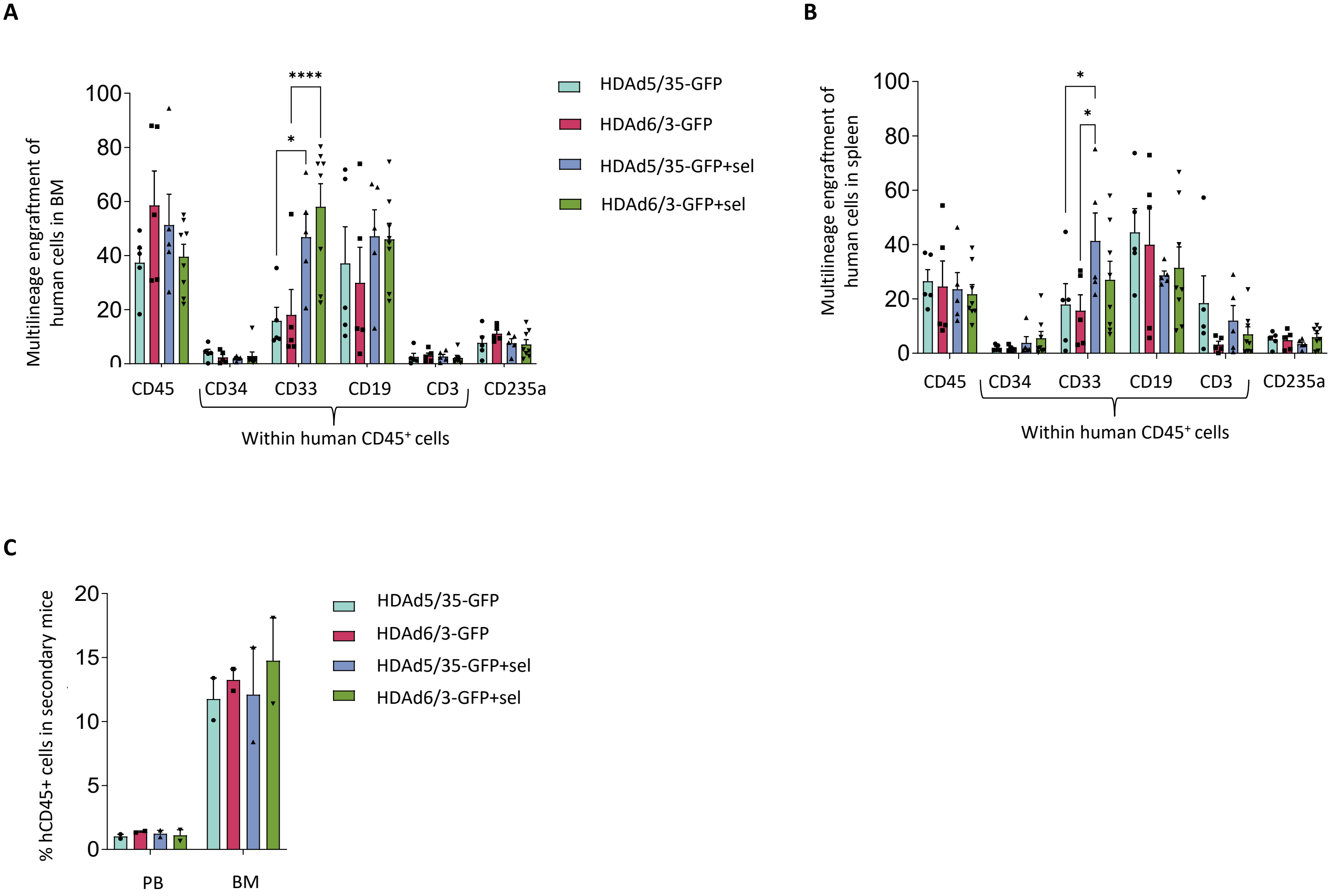

Fig.S3. Multilineage engraftment of human cells in NSGW41 mice post *in vivo* transduction with HDAd5/35-GFP or HDAd6/3-GFP. A) Multi-lineage engraftment of human cells in BM and **B)** spleen. Each symbol represents an individual mouse (HDAd5/35-GFP: N=5, HDAd6/3-GFP: N=5, HDAd5/35-GFP+sel: N=5, HDAd6/3-GFP+sel: N=8). **C)** Human chimerism in the peripheral blood and bone marrow in secondary mice. (N=8; HDAd5/35-GFP: N=2, HDAd6/3-GFP: N=2, HDAd5/35-GFP+sel: N=2 and HDAd6/3-GFP+sel: N=2). Data are shown as means ± SEM. ****p≤0.0001, *p≤0.05 (Two-way ANOVA with Bonferroni correction).

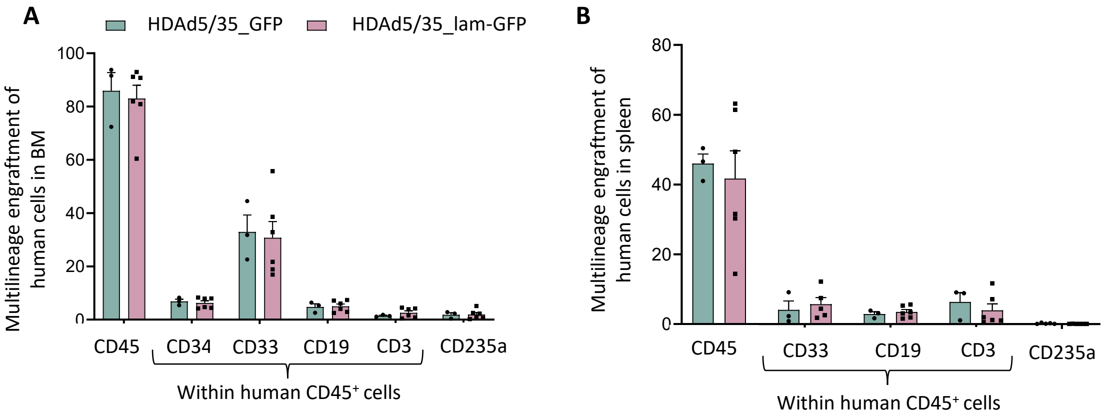

Fig.S4. Transduction efficiency of HDAd5/35_lam-GFP in human CD34⁺ cells in humanized mice, 3 days post *in vivo* transduction. Composition of **A)** BM and **B)** Spleen. Each symbol represents an individual mouse (HDAd5/35-GFP: N=3 and HDAd5/35_lam-GFP: N=6). Data are shown as means ± SEM.

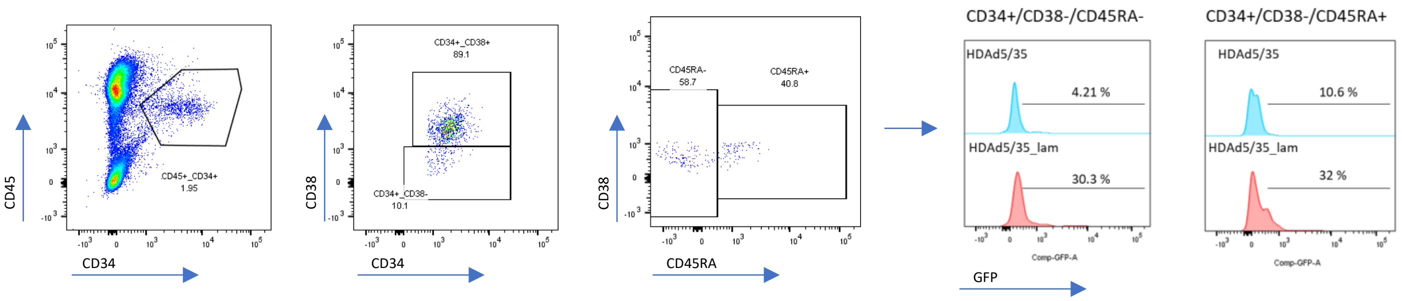

Fig.S5. Gating strategy for measuring GFP expression in HSPCs primitive subpopulations in the bone marrow of humanized mice, 3 days post *in vivo* transduction with either HDAd5/35_GFP or HDAd5/35_lam/GFP.

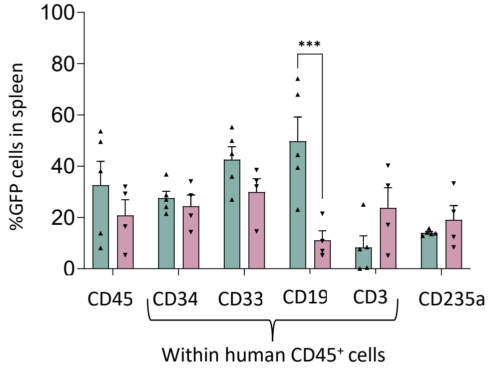

Fig.S6. Transduction efficiency of HDAd5/35_lam-GFP in hCD34⁺ cells in humanized mice, at week 15 post *in vivo* transduction. Percentage of GFP expression in human subpopulations in spleen. Each symbol represents an individual mouse (HDAd5/35-GFP: N=3 and HDAd5/35_lam-GFP: N=6). Data are shown as means ± SEM.

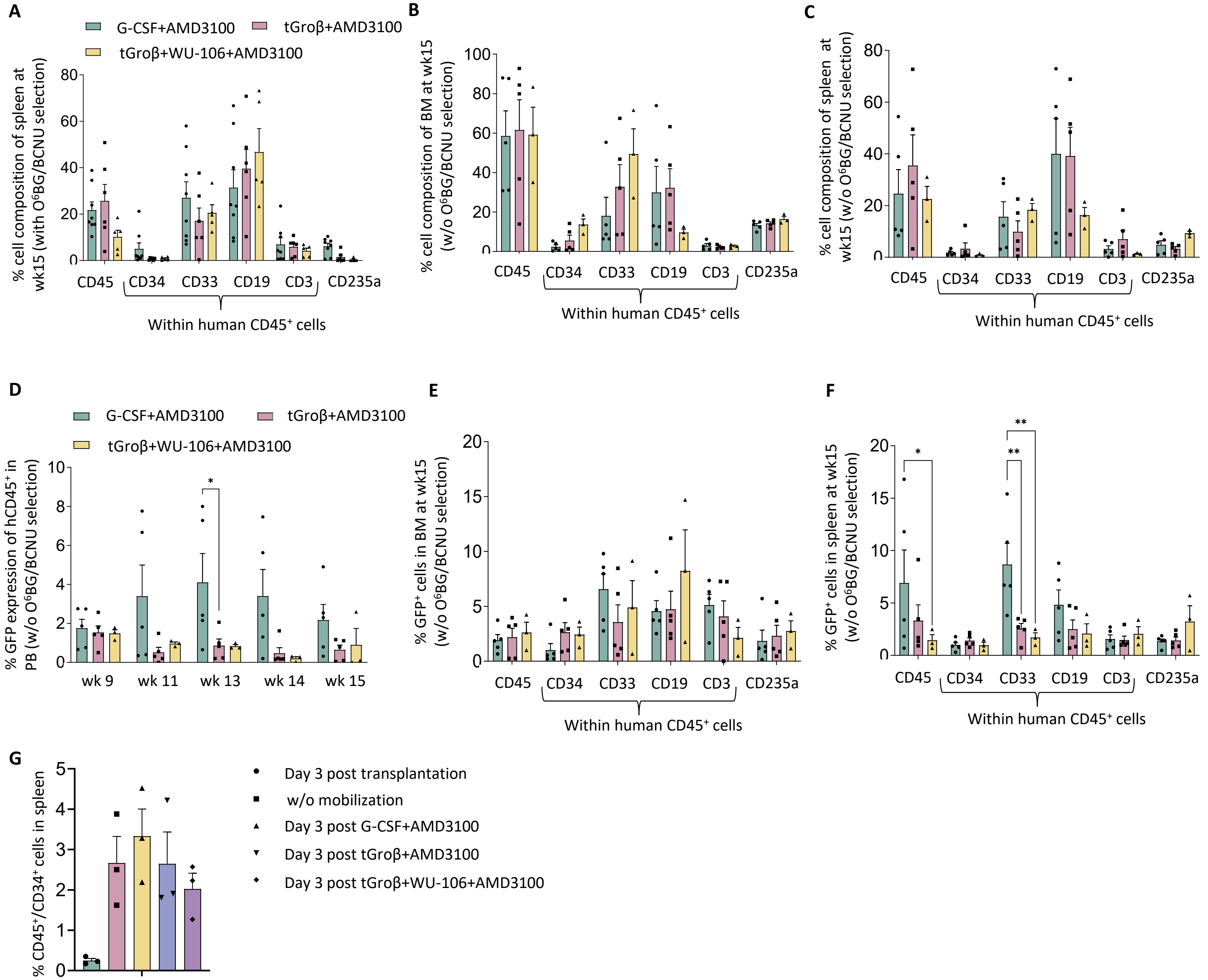

Fig.S7. *In vivo* transduction efficiency of human HSPCs with HDAd6/3-GFP mobilized with different approaches. A) Composition of human subpopulations in spleen one month after *in vivo* selection (G-CSF+AMD3100: N=8, tGroβ+AMD3100: N=6, tGroβ+WU-106+AMD3100: N=5). **Upper panel B-C)** Multilineage engraftment of human cells in **B)** BM and **C)** spleen, 8 weeks post *in vivo* transduction of mobilized human cells, without administration of O^6BG/BCNU selection drugs. **Lower panel D-F)** Percentage of GFP expressing cells in the hematopoietic tissues of mice without *in vivo* selection at week 15, two months after *in vivo* transduction of human cells **D)** in human $CD45^+$ cell in PB, **E)** in human subpopulations in BM, **F)** in human subpopulations in spleen. <u>Groups without selection:</u> G-CSF+AMD3100: N=5, tGroβ+AMD3100: N=5, tGroβ+WU-106+AMD3100: N=3. **G)** Assessment of homing of $CD34^+$ cells in spleen. Percentage of $CD34^+$ cells in spleen 3 days post transplantation of human $CD34^+$ cells, at week 6 without mobilization and 3 days post different mobilization approaches (N=3 mice per group). Each symbol represents an individual mouse. Data are shown as means ± SEM. $^{**}p \leq 0.01$, $^{*}p \leq 0.05$ (Two-way ANOVA with Bonferroni correction).

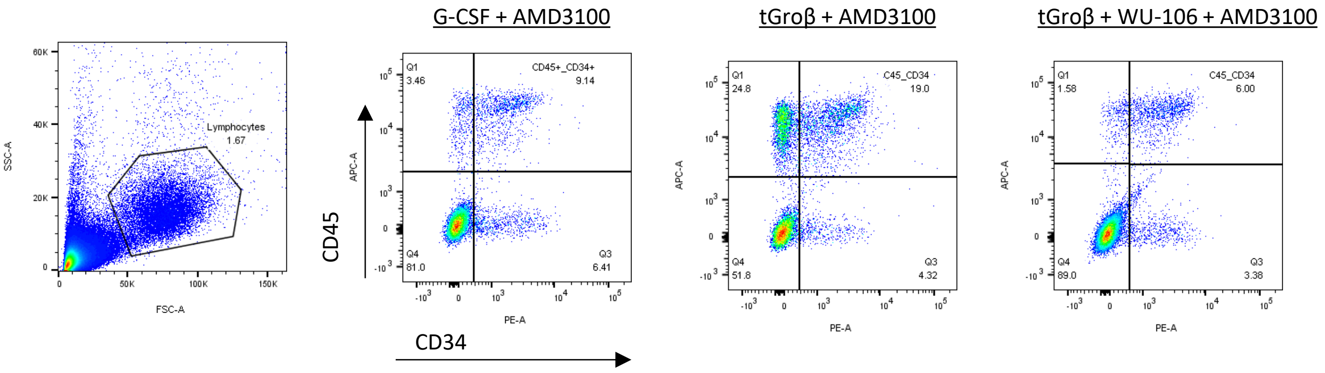

Fig.S8. A) Gating strategy for measuring the mobilization efficiency in the peripheral blood in humanized mice, at the peak of mobilization for the three mobilization schemes and representative plots of human HSCs in peripheral blood post mobilization.

Table S1. List of antibodies used for flow cytometry.

Epitope	Clone	Company	Catalog Number
**CD33**	P67.6	BD Biosciences	3058564
**CD34**	561_APC	BioLegend	343608
**CD34**	561_PE	BioLegend	343606
**CD38**	HIT2	BD Biosciences	555460
**CD90**	5E10	BD Biosciences	555597
**CD235a**	GA-R2	BD Biosciences	555570
**CD71**	M-A712	BD Biosciences	555536
**CD45**	HI30	BD Biosciences	555485
**CD19**	HIB19	BioLegend	302228
**CD3**	UCHT1	BioLegend	300406
**CD45RA**	5H9	BD Biosciences	561212
**CD46**	MEM-258	BioLegend	315304
**DSG2**	CSTEM28	ThermoFisher Scientific	12-9159-42
